# Next-generation sequencing of newborn screening genes: the accuracy of short-read mapping

**DOI:** 10.1038/s41525-020-00142-z

**Published:** 2020-09-04

**Authors:** C. Trier, G. Fournous, J. M. Strand, A. Stray-Pedersen, R. D. Pettersen, A. D. Rowe

**Affiliations:** Department of Newborn Screening, Division of Paediatric and Adolescent Medicine, Oslo University Hospital HF, 0424 Oslo, Norway

**Keywords:** Genetic testing, Paediatrics, Genetic testing, Paediatrics, Genetic testing

## Abstract

Newborn screening programs are an integral part of public health systems aiming to save lives and improve the quality of life for infants with treatable disorders. Technological advancements have driven the expansion of newborn screening programs in the last two decades and the development of fast, accurate next-generation sequencing technology has opened the door to a range of possibilities in the field. However, technological challenges with short-read next-generation sequencing technologies remain significant in highly homologous genomic regions such as pseudogenes or paralogous genes and need to be considered when implemented in screening programs. Here, we simulate 50 genomes from populations around the world to test the extent to which high homology regions affect short-read mapping of genes related to newborn screening disorders and the impact of differential read lengths and ethnic backgrounds. We examine a 158 gene screening panel directly relevant to newborn screening and identify gene regions where read mapping is affected by homologous genomic regions at different read lengths. We also determine that the patient’s ethnic background does not have a widespread impact on mapping accuracy or coverage. Additionally, we identify newborn screening genes where alternative forms of sequencing or variant calling pipelines should be considered and demonstrate that alterations to standard variant calling can retrieve some formerly uncalled variants.

## Introduction

The expansion of newborn screening (NBS) programs is among the great achievements in public healthcare systems in the past two decades^1^. The main aim of NBS is the early diagnosis of life-threatening or debilitating disorders whose outcomes can be dramatically improved upon immediate, pre-symptomatic treatment. NBS began in the early 1960s with the development of NBS for phenylketonuria (PKU) to prevent severe intellectual disability^2,3^; and in the last 20 years has expanded to include 20+ disorders through a combination of technological advancements^4^ and improved scientific knowledge. NBS programs vary by country and predominantly include testing for a range of inborn metabolic errors, endocrine disorders, primary immunodeficiency disorders, congenital deafness, congenital heart defects and cystic fibrosis^5^. Technological advances such as tandem mass spectrometry^6^ and genetic sequencing^7,8^ have thus far formed the technological basis of blood sample dependent NBS programs, and the imminence of relevant gene therapies and routine use of next-generation sequencing (NGS) in clinical laboratories means that significant opportunities for NBS have arisen.

The NBS process typically entails metabolic and/or genetic analysis of dried blood spots taken within the first few days of life. Most commonly, a multi-tier system is implemented where a first-tier metabolic analysis is performed, and further confirmation is sought for abnormal results through repeated, more fine-tuned metabolic testing. Depending on the laboratory and suspected disorder, follow-up genetic analysis may be performed by sequencing of the relevant gene/genes on DNA extracted from the dried blood spots. It is imperative that the screening process is performed quickly, as rapid diagnosis can save lives. With the decreasing costs of NGS technology, wide-scale implementation for confirmatory testing is becoming an alluring possibility for NBS^9^. Through its massively parallel capacity, NGS allows for the rapid analysis of genomic data on a large scale. It is now feasible to analyze numerous genes associated with heterogenous genetic disorders from many patients simultaneously, an approach which would be prohibitively labor intensive and costly with traditional Sanger sequencing. Hence, NGS is capable of quickly generating a large amount of genomic data that can be examined to identify pathogenic mutations, which would be an asset for NBS^9,10^ and allow for further expansion of programs to include additional disorders that can be diagnosed and treated presymptomatically^11^. NGS is increasingly being utilized in clinical diagnostics through targeted gene panels, whole-exome sequencing (WES) and whole-genome sequencing (WGS), and can even be performed on DNA extracted from the dried blood spots^12^. NBS programs have also begun to integrate NGS technology for genetic analysis to confirm diagnoses^10^. As the cost-effectiveness of NGS technology continues to rise, its use is being suggested for NBS at the whole population level^13–16^.

Though decreasing costs and improved accuracy and efficiency of genome sequencing technologies have ushered in a new era of clinical diagnostics, NGS technology is not without its shortcomings. It is therefore critical moving forward that the technical challenges associated with NGS technology are taken into account when used for clinical verification and diagnostics in NBS programs. The most common form of NGS used in clinical laboratories is short-read NGS, which to date is more accurate than long-read NGS^17^. Yet, one of the major challenges of short-read NGS is that short reads may be difficult to place in a genomic context. As their sequences are by nature short, regions with repeat sequences or of high homology in the genome are particularly problematic since they cannot be uniquely mapped to a reference genome. Paralogous genes or pseudogenes therefore present a challenge, as short reads may not uniquely map to the correct gene of interest^18^. Consequently, incomplete coverage or mismapping of reads in the genome may occur, potentially leading to false negative or positive diagnoses if not handled carefully.

There are a number of factors that may influence mapping quality and accuracy to genes of interest in NBS, particularly in the presence of high homology regions. For instance, some of the genes related to NBS disorders are in highly variable areas of the genome where alternate scaffolds have been created to account for haplotype diversity in the current human genome assembly^19^. Thus, genetic diversity not associated with pathogenic variation may affect read mapping, and the accuracy of diagnoses may depend on how similar a given individual is to the reference genome. Additionally, the length of short reads being sequenced is also expected to affect the extent of the problem homologous regions pose. Longer reads have been shown to improve mapping in homologous regions^20^, but the impact to which differing read lengths may affect sequencing results of the selection of genes included in an NBS gene panel has to our knowledge yet to be tested. Hence, highlighting areas that may be problematic and understanding the best ways to treat them is essential moving forward with NBS in the era of NGS data.

Here, we first identify high homology regions that may affect diagnosis during NBS with the use of short-read NGS. We then examine the effect homologous regions may have on clinical diagnoses by assessing mapping performance with differing NGS read lengths and patient’s ethnic backgrounds. Furthermore, we test multiple variant calling strategies on a NBS gene with extensive homology to a pseudogene to determine whether difficulties with pseudogene homology can be overcome with adjustments to the bioinformatic pipeline.

## Results

### Identification of homologous regions to NBS exons

**Fig. 1:**
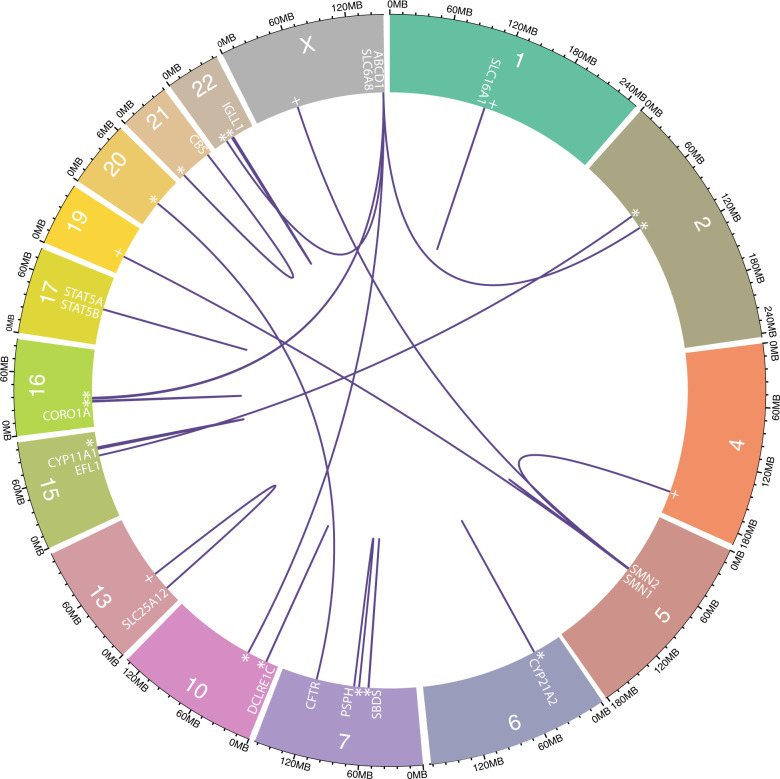
NBS genes with highly homologous regions elsewhere in the genome. The chromosomal position of all NBS genes with exonic regions sharing high homology fragments with pseudogenes (*), intergenic regions (+) or paralogous genes.

BLAST+^21^ analysis of NBS exons identified widespread homology with 525 matches of exonic regions to other areas of the genome when filtered for ≤10 mismatches and a difference in alignment length ≤10 (Supplementary Table 1), identifying 17 genes as most problematic for short read mapping (Fig. 1). The 75 k-mer CGR Alignability track identified 141 genes with exonic regions with mappability values ≤0.5. The results of the BLAST+ analysis and alignability track which were combined to conservatively include all NBS genes that may have regions of poor alignability, resulted in 144 NBS genes being included for simulation analyses (Supplementary Table 2).

### Population structuring and differentiation

**Fig. 2:**
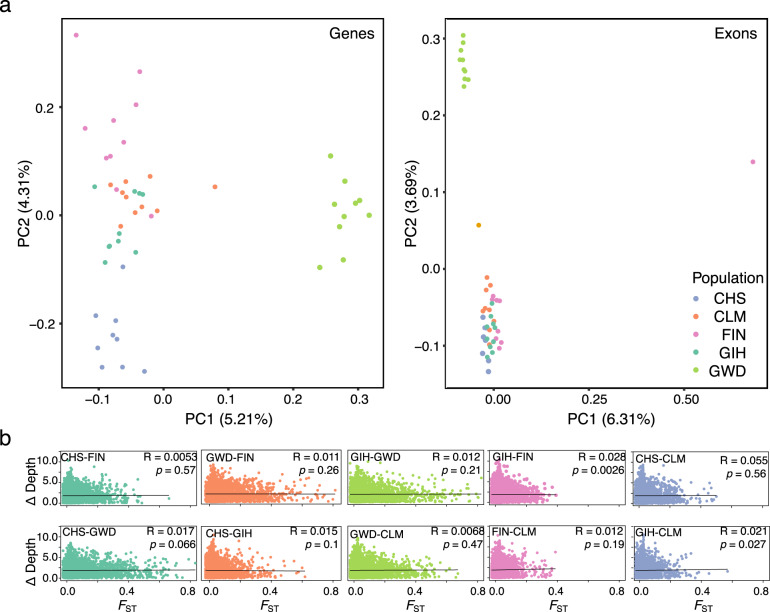
Population structuring and the effect of population-specific genetic variation on read mapping coverage. **a** PCA analysis of genetic variation in each simulated individual of the five tested populations across all NBS genes (left) and exonic regions of NBS genes (right) for 150 bp read lengths. **b** The difference in mapping depth for 1 kb stepping genomic windows across all NBS genes between two tested populations plotted against pairwise *F*_ST_ values for the same genomic windows and populations.

Principal component analysis (PCA) revealed evidence for population structuring among the mapped reads of simulated individuals indicating that there is genetic variation associated with differing ethnic backgrounds in the NBS genes. The Gambian (GWD) population separated from the other populations on PC1 and the Southern Hahn Chinese (CHS) population separated on PC2 (Fig. 2a). However, when mapped reads were filtered for only exonic regions, a single individual separated out along PC1, while the GWD population separated from the other samples along PC2 (Fig. 2b). Therefore, while population-specific genetic variation is evident in NBS genes, this pattern is driven primarily by intronic regions. In exonic regions, while there is still evidence of population level structuring, the primary axis of differentiation is at an individual level.

Global *F*_ST_ estimates of population differentiation in simulated NBS genes were overall low (*F*_ST_ range: 0.047–0.165, Table 1). The highest *F*_ST_ estimates were found between the GWD population and all others (Table 1), consistent with the PCA analyses. Overall depth of mapping coverage was highly similar between all populations across all simulated NBS genes (Table 2). Furthermore, differences in mapping coverage between populations was not significantly correlated to *F*_ST_ estimates for most population comparisons, though there were weak positive correlations in the Gujarati Indian (GIH)-Finnish (FIN) and GIH-Colombian (CLM) comparisons (Fig. 2b). Together, the *F*_ST_ and depth results indicate that genetic variation from different ethnic backgrounds does not create widespread disparities in depth of coverage when mapped to the human reference genome in NBS genes. This is further supported by overall mapping accuracy which was nearly identical between populations at mapping quality (MQ) thresholds of 10 and 20 (Supplementary Table 3).

**Table 1:** Global *F*_ST_ estimates across simulated NBS genes between populations.

	CHS	GIH	GWD	FIN
GIH	0.071			
GWD	0.165	0.141		
FIN	0.104	0.058	0.152	
CLM	0.076	0.051	0.120	0.047

**Table 2:** Average depth of mapping coverage across simulated NBS genes by population.

Population	Average depth	Standard deviation
GIH	39.997	3.801
CLM	39.995	3.803
CHS	39.990	3.800
FIN	39.992	3.799
GWD	39.994	3.807

### The effect of read length on mapping accuracy

As expected, mapping accuracy and depth improved with longer reads (Table 3: One-way ANOVA; *p*-value < 2e-16, Tukey HSD all comparisons; *p*-value < 2e-16, Supplementary Table 4). With all read lengths, >99% of reads mapped correctly. However, there was a higher percentage of correctly mapped reads, fewer incorrectly mapped and fewer unmapped reads at longer read lengths (Supplementary Table 4). The average depth of coverage across simulated NBS genes also increased with read length while standard deviation decreased (Table 3).

**Table 3:** Average depth of mapping coverage across simulated NBS genes by read length.

Read length	Average depth	Standard deviation
70	38.029	4.060
100	38.214	3.594
150	38.394	3.231
250	38.636	2.929

**Fig. 3:**
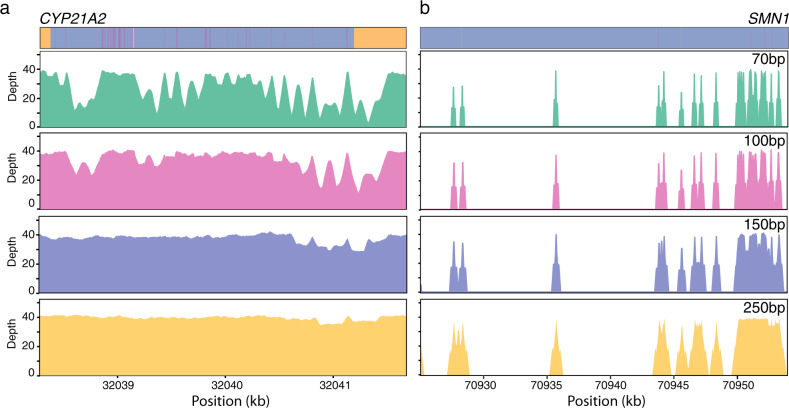
The effect of read length on mapping coverage of genes with high homology regions. Depth of read mapping coverage for different read lengths across *CYP21A2 (***a**) and *SMN1* (**b**). Sequence alignments of *CYP21A2* to *CYP21A1P* (top left) and *SMN1* to *SMN2* (top right) are depicted with indels in yellow, mismatches in pink and identical bases in purple.

There were 43 NBS genes with low depth regions below 20X once reads were filtered for a MQ ≥ 20. Of these genes, the impact of longer read lengths was dependent on the extent of homology to regions outside the gene. Therefore, there were 35 genes that had low depth regions with the shorter read lengths which were remedied by longer read lengths (Fig. 3a and Supplementary Table 5). Moreover, there were eight NBS genes with large regions of high homology which 250 bp read lengths were unable to span, leading to low coverage due to nonspecific mapping (Fig. 3b and Supplementary Table 5). Of the genes with low coverage, ten had low coverage regions within exons, and of the eight genes with low coverage across all read lengths, four had low coverage exonic regions (Supplementary Table 6). We found that the genes that had low coverage exon regions across all read lengths all had a high degree of similarity to another genomic region with zero mismatches and very few differences in alignment length according to the BLAST+ results, when compared to the other simulated genes (Supplementary Table 1). This suggests that degree and length of homology are the key factors impacting mapping success.

The four genes found to have low coverage regions within exons at all read lengths are *SMN1*, *SMN2, CBS*, and *CORO1A*. *SMN1* and *SMN2* are two paralogous genes well known to be problematic for sequencing and mapping as they are nearly identical^22,23^. Deletions encompassing exon 7 in *SMN1* are the most frequent molecular cause of spinal muscular atrophy (SMA), while the number of *SMN2* copies has been associated with severity and onset time of SMA^24–26^. *CBS* deficiency is the most common cause of homocystinuria, and the biochemical screening marker used as first-tier screening test is methionine^27^. *CBS* is used to confirm homocystinuria when high levels of methionine are detected by mass spectrometry. *CORO1A* is included in the NBS panel as it is one of the multiple genes related to severe combined immunodeficiency (SCID)^28–30^, and panel tested on the DNA extract when first-tier screening for SCID detects zero or low levels of T cell receptor excision circles (TRECs) by qPCR quantification^7^. The mapping results for these four genes containing the most problematic exonic regions with low mapping coverage are consistent with existing genomic data in gnomAD (.v3), which includes sequencing and mapping data from 71,702 whole genomes (https://gnomad.broadinstitute.org/). For each of the low coverage exon regions we identified, we find that gnomAD reports a mean coverage below 10X across most of the region in contrast to ~30X throughout most of the genome. Consequently, these genes can only be successfully NGS sequenced and mapped with an alternate strategy to standard short-read NGS workflows.

For each read length, we generated BED files that can be used as a resource indicating NBS gene regions potentially camouflaged by high homology regions and indicate where they may alternately map in the genome based on our simulation analyses (https://github.com/cntrier/NBS_short-read_mapping_paper/tree/master/Problem_Region_Bed_Files/Final_Bed_Files; also see Supplementary Tables 7–10 for lists of low coverage regions at each read length).

### Pathogenic variant calling on *CYP21A2*

**Fig. 4:**
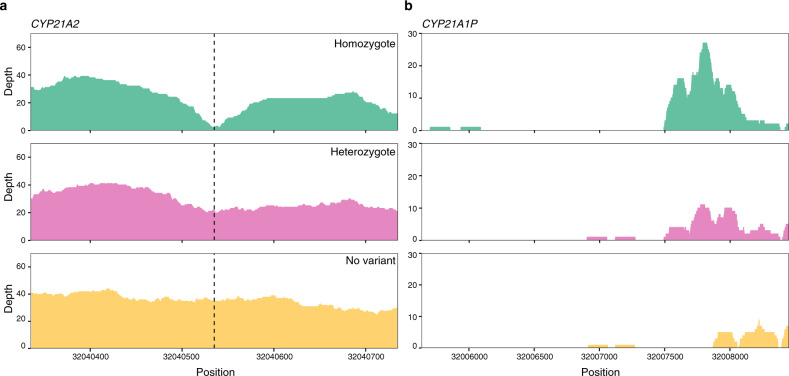
The effect of a known pathogenic variant on read mapping coverage of *CYP21A2*. Depth of read mapping coverage on *CYP21A2* (**a**) and its pseudogene *CYP21A1P* (**b**) that reads alternatively map to when no variant, a homozygous variant and a heterozygous variant (variant position indicated by dotted line) is present in *CYP21A2*.

Despite extensive homology with *CYP21A1P*, variant calling of pathogenic variants on *CYP21A2* at 150 bp read lengths was accurate for 10 out of 11 simulated single nucleotide polymorphisms (SNPs) with default GATK^31^ HaplotypeCaller settings for both homozygous and heterozygous variants (Supplementary Tables 11 and S12). This is consistent with the results from depth analysis that found no bases in *CYP21A2* with depth <20 at 150 bp read lengths (Supplementary Table 5). There was however one pathogenic SNP variant on *CYP21A2* that was not called by HaplotypeCaller when the MQ parameter was set to the default 20 or lowered to 10 (Supplementary Tables 11–13). Inspection of depth of coverage across the region, revealed that once the pathogenic variant was inserted, reads in the vicinity mapped to the pseudogene as well, lowering the coverage below the threshold for detection (Fig. 4). This indicates that some regions of NBS genes affected by pseudogene homology are sensitive to a very small amount of variation, thereby reducing the chance of variant detection in the region. When homologous regions in *CYP21A1P* were masked and *CYP21A2* was variant called with a ploidy of four as suggested in Ebbert et al., the variant was called with the genotype 1/1/1/1 (Supplementary Table 14). Therefore, masking regions of the pseudogene and increasing the ploidy during variant calling can recover variants that cause reads to map to the pseudogene once introduced if sequencing depths are stable in the sample. However, this also creates many extra variants in *CYP21A2* (Supplementary Table 15) and should only be used as a secondary means of variant calling when no variant could previously be found. In this instance, the variant formerly lost was the only called variant with a 1/1/1/1 making it easy to distinguish from variants only found in the pseudogene which had 0/0/1/1 genotype (Supplementary Table 15).

Additionally, increasing the inner distance between read pairs also recovered the initially uncalled variant and correctly variant called all other inserted pathogenic variants (Supplementary Table 16). Since BWA-MEM maps paired-end reads jointly, if one read does not map to the genome uniquely, a uniquely mapped mate read will rescue the pair, mapping both to the best alignment^32^. Therefore, if one read maps to both the NBS gene and its pseudogene while the mate maps uniquely to the NBS gene, both reads will be given a higher MQ score on the NBS gene than the pseudogene. Elongating the inner distance could lead to reads being sequenced outside of the high homology region rescuing the nonspecifically mapped mate and resulting in higher coverage of the homologous region. We found this to be the case as the read mapping depth with a MQ filter ≥10 was higher with the longer inner distance compared to the shorter (Fig. 4 and Supplementary Fig. 1).

### Variant simulation and calling on low coverage genes

None of the simulated variants in low coverage regions of *SMN1*, *CBS*, and *CORO1A* were successfully variant called using the standard GATK^31^ variant calling pipeline using the default settings, or when the inner distance between read pairs was increased. When homologous regions were masked and variant calling was performed with increased ploidy, three of the 11 simulated variants were called (Supplementary Table 17). Of these three called variants, additional variants were also called in other genomic positions. In addition, one variant that was not called generated calls at other locations (Supplementary Table 17).

## Discussion

High homology genomic regions are problematic for short-read mapping as the reads cannot be mapped uniquely to the reference genome. Efforts have been made previously to determine which areas of the human genome are highly homologous, likely presenting a challenge for short-read mapping^18,20^; yet there has been no detailed analysis of NBS-specific genes to examine how clinical diagnoses may be affected. As clinical laboratories are now routinely implementing NGS for diagnostics^33^ and it is being gradually integrated into NBS laboratory algorithms, the technical challenges associated with NGS need to be assessed and addressed.

Here, we perform a detailed analysis mapping the landscape of bioinformatic challenges associated with genes related to disorders relevant for NBS programs. We examine which regions are problematic given different short-read lengths while incorporating a range of background genetic variation. This serves to highlight which genes/regions may need to have alternative strategies in place to achieve accurate genetic diagnoses. We demonstrate that there are NBS genes containing regions with high homology to pseudogenes or paralogous genes which result in reads not mapping uniquely to these regions, causing low mapping coverage. Low coverage can make it difficult to call variants as variant calling softwares rely on mapping coverage. This could result in false negative results because a variant simply could not be identified. With this in mind, we examined the effect of different ethnic backgrounds and read lengths on read mapping of these NBS genes in the presence of high homology regions.

Different ethnicities have population-specific genetic variation^34^ which should be taken into account when designing screening programs for the general population. For this panel of NBS genes, we found little evidence that ethnic background has an effect on short-read mapping. The PCA analyses revealed evidence for population level structuring of genetic variation across NBS genes in their entirety, as well as when only exons were considered, however the main axis of differentiation was on the individual level for exonic reads. We did not find evidence for large disparities in the mapping accuracy or depth in NBS genes between populations, and little to no correlation between population differentiation and differences in mapping coverage. This suggests that in the selected genes for the NBS panel, population-specific genetic variation is small enough that it does not produce differences in read mapping on a large scale. This may be due to selective pressure against variation in disease related genes, or simply that the sample size simulated did not allow for all possible population-specific variation to be taken into account. It is also noteworthy that the genetic variation in this study is likely an underestimate as any variants that greatly affect mapping to the reference genome may not have been correctly variant called in 1000 Genomes Project which we used to simulate reads. However, the very similar mapping coverage and accuracy across all populations, even in regions with high levels of population differentiation, suggests that BWA-MEM is a robust enough mapping software to handle population-specific variation in NBS genes. While we did not detect a widespread effect of ethnic background on mapping performance, it will still be important to account for population-specific variation when designing NBS gene panels and evaluating variants. Also, the fact that mapping in high homology regions may be affected at a finer scale should be considered.

The extent of the problem high homology regions pose for short-read mapping of NBS genes is dependent on the size of homologous regions and the degree of similarity. As expected, longer read lengths improved mapping coverage and accuracy across NBS genes as they were better able to span the homologous regions. However, some NBS genes have homologous regions so large that 250 bp short-reads are not sufficiently long. We identified which genes have regions with reduced mapping coverage at each tested read length making it easier to plan an appropriate NGS sequencing strategy. For most of the NBS genes, satisfactory coverage can be achieved with standard 150 bp read lengths. In the cases of NBS genes with very large homology regions, ample mapping coverage with NGS will require an alternate strategy to standard short-read sequencing.

Currently, qPCR is used as an initial first-tier screening test for SMA. Whenever *SMN1* exon 7 is found to be deleted in the sample, quantification of *SMN2* with another method such as digital droplet PCR is performed on the same sample. To be able to properly identify and quantify *SMN1* exon 7 and *SMN2* dosage in the same run using a universal NBS gene panel would be desirable, but is not yet achievable.

Third generation long-read sequencing technologies have been shown to successfully cover large homologous regions such as in paralogous *SMN1* and *SMN2* genes^20^, yet they have notoriously high error rates which are not yet suitable frontline clinical diagnostics. However, the accuracy of long-read sequencing is continually improving as well as the tools for handling long reads. Accurate variant calling on consensus sequences of long reads can be achieved^35,36^ and may be an avenue to explore in NBS laboratory algorithms. A more labor-intensive long-range PCR strategy uniquely targeting the gene of interest and subsequent NGS short-read sequencing and mapping solely to the NBS gene has alternatively been shown to overcome problems caused by pseudogene homology^37^.

While nonspecific mappings due to high homology regions can be largely remedied by the use of longer reads in many instances, this does not necessarily mean these regions are free from issues. *CYP21A2* is the gene for 21-hydroxylase deficiency which is the most common cause of congenital adrenal hyperplasia (CAH)^38^. CAH is included in NBS since it can be life-threatening within a few days of birth in its severest clinical forms. First-tier screening for CAH is based on immunoassay measurements of 17-hydroxyprogesterone, the steroid precursor proximal to the defect. Whenever 17-OHP is elevated in the sample, further measurement of other steroids in the related pathways (21-deoxycortisol, 11-deoxycortisol, cortisol, androstendion and their ratios) are performed as secondary tests on the same dry blood spot. We show that inserting a known pathogenic variant in *CYP21A2* makes reads more similar to a pseudogene *CYP21A1P* since the pathogenic variant is the same as the pseudogene sequence, leading to the reads mismapping to the pseudogene. In this instance, lowering the mapping coverage threshold in the variant calling pipeline was not enough to successfully call the variant. A work-around has been suggested that includes the masking of homologous regions in the pseudogene and variant calling with increased ploidy^20^, which we found to be successful for this variant in *CYP21A2* and in three of the 11 simulated variants within low coverage exonic regions we identified in *SMN1* and *CBS*. Therefore, this may be a supplementary method to implement if standard variant calling cannot successfully identify a pathogenic variant for a disease suspected based on first-tier analyses or symptoms. In general, masking of pseudogenes during mapping does not solve mapping issues associated with high homology regions because primers and probes may bind to both the functional genes and the pseudogenes^39^. This could easily create false positives, or mask true variants due to effectively increased ploidy making molecular analysis unreliable. Indeed, the masking of the pseudogene in our simulation analyses created additional variants in all but one of the recovered variants. Since *CYP21A2* and its pseudogene were both homozygous for the same variant, it was the only variant with a 1/1/1/1 genotype making it easy to isolate among variants only present in the pseudogene. However, this was not the case for *SMN1* and *CBS* indicating that interpretation of variants may be difficult with this method if many additional variants are called.

While we evaluated variant calling by genotyping single samples individually, joint variant calling of multiple samples may improve its genotyping accuracy by identifying true variation when it is observed across multiple individuals in a cohort^40^. However, as NBS diseases are rare, the likelihood that the same pathogenic variant would be observed in multiple samples during a sequencing run is small and therefore improvement of accuracy is likely limited and conversely, it is possible that rare variant detection may be hindered. Multi-sample variant calling does however have the added benefit of ensuring all sites are reported in the output making it possible to distinguish from homozygous reference and missing data which would prove helpful in interpreting variant calling results.

The level of uncertainty around the type of genetic variation that may be encountered and how this will affect short-read mapping in the presence of high homology genomic regions, underscores the importance of the multi-tier system in NBS programs. It has been shown that solely relying on NGS sequencing in NBS results in fewer true positives detected and a higher number of variants of unknown significance (VUS) than the multi-tier metabolic analysis^9^. Additionally, biochemical and molecular analyses were found to be complementary^9^. Our results from variant calling on *CYP21A2*, *SMN1*, *CBS*, and *CORO1A* demonstrate that an accurate diagnosis would not have been made with NGS sequencing alone. Only a known suspected diagnosis based on first-tier analyses could direct further supplementary methods of variant calling in the specific gene to recover the lost variants. Though this study focuses on single nucleotide variants, it is also important to consider that structural variants, such as insertions, translocations and inversions, which can also be difficult to map to the reference genome and variant call^41^, could easily be missed with NGS sequencing alone. Additionally, as read lengths increase, novel variants will be found upon sequencing^42,43^, potentially confounding diagnoses and first-tier biomarker analyses may help determine if such variants in NBS genes result in a disease phenotype. Thus, multi-tier testing adds a higher level of certainty to molecular diagnoses.

It is also important to note that our simulation analyses represent a best-case scenario which does not account for various errors that may be encountered depending on the sequencing strategy and methods. In this study, we simulated uniform coverage across NBS genes for the sake of comparison but in practice if targeted amplicons are used in sequencing, coverage is rarely uniform across amplicons^44^ which greatly exacerbates problems associated with homologous regions. A capture bias of high GC exons is also expected to result in differential sequencing coverage^45^, potentially affecting analyses. With the continued decrease in the cost of NGS, WGS may become a better and more cost-effective alternative in the future as it allows for more uniform coverage^46^. In fact, WGS has been shown to overcome issues with variant detection attributed to pseudogene homology in polycystic kidney disorder through its elimination of capture bias and uniform coverage at 150 bp read lengths^47^. WGS would also provide many opportunities for the expansion of NBS. NGS strategies typically focus on exons of genes, yet it is becoming increasingly apparent that deep intronic, intergenic regulatory elements, copy number variants and other structural variants can play a significant role in different diseases^48–50^. Enabling the investigation of introns and structural and copy number variation would enhance the sensitivity of NGS and provide more flexibility for further expansion of the NBS program to encompass disorders lacking first-tier biomarkers. However, WGS would also present a new set of challenges including a high demand on computational and bioinformatic resources, sensitive data security and ethical considerations related to identification of VUS and incidental findings in newborns without clinical phenotypes. Overall, NGS provides many exciting opportunities for the improvement and expansion of NBS programs, but during its integration it will be important to keep in mind its shortcomings to ensure that screening programs are designed appropriately.

## Methods

### Data collection and BLAST+ analysis

The panel of 158 NBS genes investigated in this study is comprised of 152 genes used in the early 2017 newborn genetic screening panel at Oslo University Hospital, as well as six genes not included in the screening panel but with indications of NBS interest (Supplementary Table 18). The Oslo University Hospital newborn genetic screening panel currently screens for 25 disorders nation-wide (Supplementary Table 18) and was customized to include genes associated with disorders from the Recommended Uniform Screening Panel (RUSP) (https://www.hrsa.gov/advisory-committees/heritable-disorders/rusp/index.html), their differential diagnoses, as well as disorders likely to be included in the NBS program in the near future. While some screening disorders are related to multiple genes and can follow multiple traits (such as SCID with autosomal recessive, X-linked, autosomal dominant *de novo* occurrence), the majority of the inherited metabolic disorders are autosomal recessive and one homozygous pathogenic variant in any one of the associated genes can be found causal. A BED file of exonic positions for each gene was retrieved from GRCh38 on the Ensembl database Release 94^51^ using biomaRt^52^ (v.3.8) and a 70 bp buffer region was added up- and down-stream from each exon.

To identify genomic regions highly homologous to exonic sequences of the NBS genes, a BLAST+^21^ (v.2.8.1) analysis was performed. The human reference genome GRCh38.p12 (RefSeq accession GCF_000001405.38) was downloaded from NCBI^53^ and was first made into a repeat masked database using dustmasker^54^. FASTA sequences were retrieved for all NBS exons using BEDTools^55^ (v.2.17.0) -getfasta function from GRCh38.p12. The FASTA sequences were then each queried against the repeat masked human genome database with BLAST+ using the default settings. The locations of BLAST hits that were not the query sequence itself were recorded including the length of the match and number of mismatches from the query.

The 75 k-mer CGR Alignability track, which displays the extent to which 75 k-mer sequences uniquely align to a genomic region was downloaded from the UCSC depository (http://rohs db.cmb.usc.edu/) in BigWig format and converted to a BED file with mappability values using UCSC’s bigWigToWig tool followed by BEDOPS^56^ (v.2.4.35) wig2bed. Mappability values were binned into the coordinates of NBS exons and an average mappability value of each exon was calculated. An exon was conservatively considered potentially problematic for short-read mapping if the mappability index was <0.5 or if there was an alternate hit in the BLAST analysis with ≤10 mismatches and a difference in alignment length ≤10.

### Read simulation of 50 genomes from around the world

Read simulation of the NBS genes was performed to test the unique mappability of each potentially problematic region at differing NGS read lengths using genomes with a representative variety of ethnic backgrounds. Variant calls of 50 unrelated individuals from each of the five super populations sequenced and variant called by the 1000 Genomes Project^34^ were retrieved (Finland (FIN) = 10 N, Gambia (GWD) = 10 N, Colombia (CLM) = 10 N, Gujarati Indian in Houston TX (GIH) = 10 N, Southern Hahn Chinese (CHS) = 10 N; Supplementary Table 19) (http://ftp.1000genomes.ebi.ac.uk/vol1/ftp/data_collections/1000_genomes_project/release/20181203_biallelic_SNV/). Only females were chosen as there are no NBS genes on the Y chromosome, and this allowed for equal sample sizes among chromosomes.

A FASTA reference genome for each individual was created using bcftools^57^ (v.1.9) consensus with the VCF file for the individual and the GRCh38.p12 reference genome from the 1000 Genomes Project FTP site (ftp://ftp.1000genomes.ebi.ac.uk/vol1/ftp/technical/reference/GRCh38_reference_genome/) as input and IUPAC coding for heterozygotes. Two FASTA files were then made for each individual with each biallelic IUPAC base converted to its corresponding bases in the two FASTA files, enabling proper simulation in the next stage. Illumina paired-end reads 70, 100, 150 and 250 bp in length with an inner distance of 50 ± 10 bp were simulated using DWGSIM (v.0.1.11) (https://github.com/nh13/DWGSIM) for each FASTA file of every individual at 20X coverage with a 0.0024 error rate, no mutations or indels, and allowing for ≤5 N’s in each sequence. Reads were simulated for every gene with an exonic region identified as potentially problematic previously in the BLAST+ and GEM analyses and an extra 2 kb flanking sequence was added up- and downstream allowing for equal coverage of the differing read lengths at region boundaries. The error rate was chosen based on recent empirical estimates of next-generation sequencing error rates^58^. The two read sets for the same sequence length and individual were then combined for a total coverage of 40X per individual.

### Alignment of simulated reads and post-processing

The simulated reads were mapped to the GRCh38.p12 human reference genome from the 1000 Genomes Project which includes alternate contigs, unplaced and unlocalized scaffolds and decoy sequences using BWA-MEM^32^ (v.0.7.17). BWA-MEM^32^ (0.7.17) performs alternate scaffold aware (ALT-aware) mapping by default which allows for multiple mappings of a read to the primary assembly and alternate contigs with prioritization to the primary assembly.

Mapping to the human reference genome was run with the following command:

bwa mem -t 1 -B 4 -O 6 -E 1 -M reference_fasta_file fastq_R1 fastq_R2 | samtools sort | samtools view -1 - > bam_file

Results from the read simulation were evaluated using the dwgsim_eval script provided by DWGSIM (https://github.com/nh13/DWGSIM), with the -p option to identify incorrect alignments and the -a 0 option to output mapping quality and overall incorrect/correct read counts. Results were evaluated separately for each population and read length for comparison, as well as in combination. Reads with a MQ < 10 were considered unmapped as they would typically be filtered out in downstream analyses. The depth of all simulated regions was calculated from the BWA output bam files using samtools depth (http://www.htslib.org) with the -q 9 and -a parameters. Depth was calculated for each population and read length separately for comparison and in combination for overall values.

### Population structuring and differentiation

To test if there is evidence for population structuring among the simulated regions, a PCA analysis was run for the 150 bp read length library of each simulated individual. First, genotype likelihoods were calculated from each individuals’ bam file using ANGSD^59^ (v.0.918) with the following command:

angsd -b bam.list -nThreads 10 -out $outfile_name -GL 2 -doMaf 2 -doMajorMinor 1

-doGeno 32 -doPost 1 -SNP_pval 1e-3 -nind 50 -P 8

The covariance matrix of the genotype likelihoods was calculated using ngsTools’^60^ (v.3) ngsCovar tool with -nsites set to 100000. The PCA plot was created using the plotPCA.R script provided in ngsTools with the covariance file as input.

Genetic differentiation between the simulated populations across NBS genes was calculated with *F*_ST_ estimates using ANGSD^59^ (v.0.918) with the 150 bp read length libraries. Per-site *F*_ST_ values were binned into 150 bp stepping windows with ANGSD’s *F*_ST_ window function with -type 0 ensuring the genomic windows were identical between population comparisons. To test if there was a significant correlation between population divergence and mapping coverage, which would indicate that genetic variation associated with ethnic background affects mapping coverage, a Spearman correlation was performed between the 1 kb binned *F*_ST_ estimates across NBS genes and average mapping coverage values binned into the same 1 kb bins for each population pair.

### Identification of low depth regions

For each simulated read length, regions of low mapping coverage were gathered and annotated into BED files along with the alternate regions where simulated reads mapped. To do so, per-position depth calculations from samtools -depth were filtered for bases with a depth <20. Consecutive low coverage bases were combined into a single larger region if they were within 50 bases of each other using BEDTools^55^ (v.2.17.0) merge -d 50. Regions were annotated using annotation information from the RefSeq GRC38.p12 annotation file (GCF_000001405.38_GRCh38.p12_genomic.fna.gz) using BEDOPS^56^ bedmap (2.4.35). The alternate regions that simulated reads mapped to were extracted from the dwgsim_eval -p -q 0 output analysis of bam files. Each region was also annotated using BEDOPS^56^ bedmap (v.2.4.35) and was merged in the low coverage region BED file with the corresponding region it was simulated. The total number of reads for each low depth region, as well as the number of reads in each region that were not uniquely mapped (MQ < 10) were calculated using a custom script (See Code Availability).

### Pathogenic variant simulation and variant calling on *CYP21A2*

Sequence similarity between *CYP21A2* and *CYP21A1P* and paralogous genes *SMN1* and *SMN2* was calculated by first running a MUSCLE pairwise alignment in Geneious (v. 2019.1.3) (https://www.geneious.com) under the default settings. The exported alignment file was plotted using AlignFigR (https://github.com/sjspielman/alignfigR) followed by custom alterations.

To assess how clinical diagnosis of NBS genes may be affected by highly homologous sequences in the genome, pathogenic variants on *CYP21A2* which shares 97.7% sequence homology with its pseudogene *CYP21A1P* were simulated and variant calling was performed. First, a VCF file of human variants and disease associations was downloaded from ClinVar^61^ VCF (v.20181217). The VCF file was filtered for pathogenic variants within exonic regions of *CYP21A2* using VCFtools^62^ (v.0.1.13). A homozygote and heterozygote VCF file was created for each pathogenic variant. Additionally, a random individual (HG02763) was selected to serve as the reference for pathogenic variant simulation so that non-disease related human variation could also be incorporated in the analysis. Using bcftools^57^(v.1.9), each pathogenic variant VCF was applied to the reference FASTA to create a consensus FASTA with IUPAC coding. DWGSIM (v.0.1.11) (https://github.com/nh13/DWGSIM) was run separately for each homozygote and heterozygote FASTA sequence for every pathogenic variant with Illumina paired-end 150 bp read lengths, an inner distance of 50 ± 10 bp, 40X coverage with a 0.0024 error rate, no mutations or indels and allowing for ≤5 N’s in the sequence. The BED file of all previously simulated NBS genes with 2 kb up- and downstream flanking sequences was provided as input to restrict regions of simulation. As DWGSIM outputs heterozygote IUPAC codes as N, the FASTQ files were altered so that half of the reads had the alternate allele and the other half had the reference allele. Simulated reads were then mapped to the GRCh38.p12 human reference genome used previously with BWA-MEM^32^ (v.0.7.17) and the same parameters as previously.

Variant calling was performed on the processed reads with GATK^31^ (v.4.0) Haplotypecaller using the -ERC GVCF parameter and subsequently genotyped with GenotypeGVCFs using the default settings. Variant calling was also performed using the same general pipeline with modifications to see if accuracy could be improved which included (1) increasing inner distance of read pairs (2) decreasing the MQ quality cutoff (3) masking homologous regions and variant calling with increased ploidy. To test for improvement with increased inner distance, the same pipeline as previously was run with the exception of the inner distance set to 255 bp in DWGSIM (v.0.1.11) (https://github.com/nh13/DWGSIM) (the largest distance the software can simulate). To decrease the MQ cutoff for reads considered in variant calling, the -mq 10 option was input when Haplotypecaller was being run. Finally, homologous regions on *CYP21A1P* identified during BLAST+ analysis of *CYP21A2* were masked in the reference genome using BEDTools^55^ -maskfasta. Reads were simulated for both *CYP21A2* and *CYP21A1P* since even when *CYP21A2* is targeted, residual amplification or capture of the pseudogene may occur and should be considered. Simulated reads were then mapped to the masked reference genome and variants called using the default settings with the exception of the ploidy being set to 4.

### Pathogenic variant simulation and variant calling on low coverage genes

In order to test how applicable the alterations to the variant calling pipeline tested on *CYP21A2* are to other genes in the NBS panel, we also tested variant calling with increased ploidy and increased inner distance on the genes we found to have low coverage exonic regions. To do so, five variants classified as pathogenic according to the ClinVar^61^ VCF (v.2020052) within regions identified with low coverage were included from both *SMN1* and *CBS* in the analysis, as well as the one variant in the low coverage region of *CORO1A* which has previously been classified as likely benign (Supplementary Table 17). Variants were simulated and variant called using the standard GATK^31^ pipeline using the default settings, with an increased inner distance and with increased ploidy and the masking of homologous regions. The same variant simulation and variant calling methodology was performed as on *CYP21A2* with the exceptions that only homozygous variants were considered, and an updated software version of GATK^31^ (v.4.1.2.0) was used. To mask the reference genome, regions homologous to the low coverage exonic regions within the gene of interest, which were identified in our mapping analysis, as well as 2 kb up- and downstream flanking sequences were masked.

### Ethical compliance

Ethical approval was not deemed necessary in this study as simulations were run from already published and publicly available data.

### Reporting summary

Further information on research design is available in the Nature Research Reporting Summary linked to this article.

## Supplementary information

Supplementary Information

Reporting Summary

## Data Availability

The datasets generated during and/or analyzed during the current study are available at https://github.com/cntrier/NBS_short-read_mapping_paper. Generated mapped read files have been deposited in zenodo.org under the DOI 10.5281/zenodo.3950369. VCF files used for read simulation are publicly available from the 1000 Genome Project at http://ftp.1000genomes.ebi.ac.uk/vol1/ftp/data_collections/1000_genomes_project/release/20181203_biallelic_SNV/.

## Code availability

All software versions and parameters needed to reproduce the results are specified in the “Methods”. Code developed to run analyses in the study is publicly available at https://github.com/cntrier/NBS_short-read_mapping_paper.

## References

[1] Centers for Disease Control and Prevention (CDC). (2011). Ten great public health achievements–United States, 2001–2010. Morb. Mortal. Wkly. Rep..

[2] Guthrie R, Susi A (1963). A simple phenylalanine method for detecting phenylketonuria in large populations of newborn infants. Pediatrics.

[3] Guthrie R (1992). The origin of newborn screening. Screening.

[4] Wilcken B, Wiley V, Hammond J, Carpenter K (2003). Screening newborns for inborn errors of metabolism by tandem mass spectrometry. N. Engl. J. Med..

[5] Therrell BL (2015). Current status of newborn screening worldwide: 2015. Semin Perinatol..

[6] Millington DS, Norwood DL, Kodo N, Roe CR, Inouet F (1989). Application of fast atom bombardment with tandem mass spectrometry and liquid chromatography/ mass spectrometry to the analysis of acylcarnitines in human urine, blood, and tissue. Anal. Biochem..

[7] Strand J (2020). Second-tier next generation sequencing integrated in nationwide newborn screening provides rapid molecular diagnostics of severe combined immunodeficiency. Front. Immunol..

[8] Lundman E (2016). Implementation of newborn screening for cystic fibrosis in Norway. Results from the first three years. J. Cyst. Fibros..

[9] Bodian DL (2016). Utility of whole-genome sequencing for detection of newborn screening disorders in a population cohort of 1,696 neonates. Genet Med..

[10] Landau YE, Lichter-Konecki U, Levy HL (2014). Genomics in newborn screening. J. Pediatr..

[11] Berg JS, Powell CM (2015). Potential uses and inherent challenges of using genome-scale sequencing to augment current newborn screening. Cold Spring Harb. Perspect. Med..

[12] Bassaganyas L (2018). Whole exome and whole genome sequencing with dried blood spot DNA without whole genome amplification. Hum. Mutat..

[13] Saunders CJ (2012). Rapid whole-genome sequencing for genetic disease diagnosis in neonatal intensive care units. Sci. Transl. Med..

[14] Francescatto L, Katsanis N (2015). Newborn screening and the era of medical genomics. Semin Perinatol..

[15] Almannai M, Marom R, Reid Sutton V (2016). Newborn screening: a review of history, recent advancements, and future perspectives in the era of next generation sequencing. Curr. Opin. Pediatr..

[16] Boemer F (2017). A next-generation newborn screening pilot study: NGS on dried blood spots detects causal mutations in patients with inherited metabolic diseases. Sci Rep..

[17] Goodwin S, McPherson JD, McCombie WR (2016). Coming of age: Ten years of next-generation sequencing technologies. Nat. Rev. Genet..

[18] Mandelker D (2016). Navigating highly homologous genes in a molecular diagnostic setting: a resource for clinical next-generation sequencing. Genet Med..

[19] Schneider VA (2017). Evaluation of GRCh38 and de novo haploid genome assemblies demonstrates the enduring quality of the reference assembly. Genome Res..

[20] Ebbert MTW (2019). Systematic analysis of dark and camouflaged genes reveals disease- relevant genes hiding in plain sight. Genome Biol..

[21] Madden TL (2009). BLAST+: architecture and applications. BMC Bioinform..

[22] Pearn J (1978). Incidence, prevalence, and gene frequency studies of chronic childhood spinal muscular atrophy. J. Med. Genet..

[23] Prior TW (2010). Newborn and carrier screening for spinal muscular atrophy. Am. J. Med. Genet A.

[24] Mailman MD (2002). Molecular analysis of spinal muscular atrophy and modification of the phenotype by SMN2. Genet Med..

[25] Swoboda KJ (2005). Natural history of denervation in SMA: relation to age, SMN2 copy number and function. Ann. Neurol..

[26] Zerres K, Wirth B, Rundik‐Schöneborn S (1997). Spinal muscular atrophy—clinical and genetic correlations. Neuromuscul. Dis..

[27] Refsum H, Fredriksen Å, Meyer K, Ueland P-M, Kase BF (2004). Birth prevalence of homocystinuria. J. Pediatr..

[28] Stray-Pedersen A (2014). Compound heterozygous CORO1A mutations in siblings with a mucocutaneous-immunodeficiency syndrome of epidermodysplasia verruciformis-HPV, molluscum contagiosum and granulomatous tuberculoid leprosy. J. Clin. Immunol..

[29] Moshous D (2013). Whole-exome sequencing identifies coronin-1A deficiency in 3 siblings with immunodeficiency and EBV-associated B-cell lymphoproliferation. J. Allergy Clin. Immunol..

[30] Shiow LR (2008). The actin regulator coronin-1A is mutated in a thymic egress deficient mouse strain and in a T-B+NK+SCID patient. Nat. Immunol..

[31] McKenna A (2010). The genome analysis toolkit: a MapReduce framework for analyzing next-generation DNA sequencing data. Genome Res..

[32] Li, H. Aligning sequence reads, clone sequences and assembly contigs with BWA-MEM. Preprint at https://arxiv.org/abs/1303.3997 (2013).

[33] Saunders CJ (2012). Rapid whole-genome sequencing for genetic disease diagnosis in neonatal intensive care units. Sci. Transl. Med..

[34] Campbell CL (2015). A global reference for human genetic variation. Nature.

[35] Koren S (2012). Hybrid error correction and de novo assembly of single-molecule sequencing reads. Nat. Biotechnol..

[36] Fu S, Wang A, Au KF (2019). A comparative evaluation of hybrid error correction methods for error-prone long reads. Genome Biol..

[37] Frans G (2018). Conventional and single-molecule targeted sequencing method for specific variant detection in IKBKG while bypassing the IKBKGP1 pseudogene. J. Mol. Diagn..

[38] Concolino P, Costella A (2018). Congenital adrenal hyperplasia (CAH) due to 21-hydroxylase deficiency: a comprehensive focus on 233 pathogenic variants of CYP21A2 gene. Mol. Diagn. Ther..

[39] Borràs DM (2017). Detecting PKD1 variants in polycystic kidney disease patients by single-molecule long-read sequencing. Hum. Mutat..

[40] The GATK Team. The logic of joint calling for germline short variants. https://gatk.broadinstitute.org/hc/en-us/articles/360035890431-The-logic-of-joint-calling-for-germline-short-variants (2020).

[41] Mahmoud M (2019). Structural variant calling: the long and the short of it. Genome Biol..

[42] Xue Y, Ankala A, Wilcox WR, Hegde MR (2015). Solving the molecular diagnostic testing conundrum for Mendelian disorders in the era of next-generation sequencing: single-gene, gene panel, or exome/genome sequencing. Genet Med..

[43] Christiaans I, Mook ORF, Alders M, Bikker H, Lekanne dit Deprez RH (2019). Large next- generation sequencing gene panels in genetic heart disease: challenges in clinical practice. Neth. Heart J..

[44] Samorodnitsky E (2015). Evaluation of hybridization capture versus amplicon-based methods for whole-exome sequencing. Hum. Mutat..

[45] Veal, C. D. et al. A mechanistic basis for amplification differences between samples and between genome regions. *BMC Genomics.* **13**, 455 (2012). 10.1186/1471-2164-13-455 PMC3469336 22950736

[46] Meienberg J, Bruggmann R, Oexle K, Matyas G (2016). Clinical sequencing: is WGS the better WES?. Hum. Genet..

[47] Mallawaarachchi AC (2016). Whole-genome sequencing overcomes pseudogene homology to diagnose autosomal dominant polycystic kidney disease. Eur. J. Hum. Genet..

[48] Li YR (2020). Rare copy number variants in over 100,000 European ancestry subjects reveal multiple disease associations. Nat. Commun..

[49] Vaz-Drago R, Custódio N, Carmo-Fonseca M (2017). Deep intronic mutations and human disease. Hum. Genet..

[50] Jutzi D, Akinyi MV, Mechtersheimer J, Frilander MJ, Ruepp M-D (2018). The emerging role of minor intron splicing in neurological disorders. Cell Stress.

[51] Flicek P (2017). Ensembl 2018. Nucleic Acids Res..

[52] Durinck S (2005). BioMart and Bioconductor: a powerful link between biological databases and microarray data analysis. Bioinformatics.

[53] O’Leary NA (2016). Reference sequence (RefSeq) database at NCBI: Current status, taxonomic expansion, and functional annotation. Nucleic Acids Res..

[54] Morgulis A, Gertz EM, Schäffer AA, Agarwala R (2006). A fast and symmetric DUST implementation to mask low-complexity DNA sequences. Comput Biol..

[55] Quinlan AR, Hall IM (2010). BEDTools: a flexible suite of utilities for comparing genomic features. Bioinformatics.

[56] Sandstrom R (2012). BEDOPS: high-performance genomic feature operations. Bioinformatics.

[57] Li H (2011). A statistical framework for SNP calling, mutation discovery, association mapping and population genetical parameter estimation from sequencing data. Bioinformatics.

[58] Pfeiffer F (2018). Systematic evaluation of error rates and causes in short samples in next- generation sequencing. Sci. Rep..

[59] Korneliussen TS, Albrechtsen A, Nielsen R (2014). ANGSD: analysis of next-generation sequencing data. BMC Bioinform..

[60] Fumagalli M (2013). Quantifying population genetic differentiation from next-generation sequencing data. Genetics.

[61] Landrum MJ (2018). ClinVar: improving access to variant interpretations and supporting evidence. Nucleic Acids Res..

[62] Danecek P (2011). The variant call format and VCFtools. Bioinformatics.

