# Supplementary material for: Next-generation sequencing of newborn screening genes: the accuracy of short-read mapping
Source: NPJ Genom Med. 2020 Sep 4;5:36. doi: 10.1038/s41525-020-00142-z (PMC7474066; doi:10.1038/s41525-020-00142-z)
Supplement: Supplementary file 1 — Supplementary Information [file 41525_2020_142_MOESM1_ESM.pdf]

Supplementary Tables and Figures

Supplementary Table 1. Results from the BLAST+ analysis.

Chr	Start	End	NBS Gene	Alignment Length	Mismatches	Hit Coordinates
X	153740504	153740674	ABCD1	0	4	chr10:38601352-38601521
X	153740504	153740674	ABCD1	0	10	chr16:32478319-32478035
X	153740504	153740674	ABCD1	0	6	chr16:32478319-32478150
X	153740504	153740674	ABCD1	0	8	chr22:16387625-16387794
X	153740504	153740674	ABCD1	0	9	chr2:91840543-91840712
X	153740504	153740789	ABCD1	0	8	chr22:16387625-16387794
X	153740504	153740789	ABCD1	0	10	chr16:32478319-32478035
X	153740504	153740789	ABCD1	0	4	chr10:38601352-38601521
X	153740504	153740789	ABCD1	0	6	chr16:32478319-32478150
X	153740504	153740789	ABCD1	0	9	chr2:91840543-91840712
X	153742917	153743141	ABCD1	0	8	chr2:91842988-91843211
X	153742917	153743141	ABCD1	0	9	chr22:16390067-16390290
X	153742917	153743141	ABCD1	0	10	chr2:89764989-89764766
X	153742917	153743141	ABCD1	0	8	chr16:32475874-32475651
X	153743151	153743416	ABCD1	5	8	chr22:16390301-16390560
X	153743151	153743416	ABCD1	5	6	chr2:91843222-91843480
X	153743151	153743416	ABCD1	5	10	chr10:38604029-38604288
X	153743151	153743416	ABCD1	2	10	chr16:32475640-32475378
21	43053121	43053717	CBS	0	0	chr21:6444800-6445395
21	43053121	43053717	CBS	0	0	chr21:6444800-6445731
21	43053121	43054053	CBS	0	0	chr21:6444800-6445395
21	43053121	43054053	CBS	0	0	chr21:6444800-6445731
21	43053685	43054053	CBS	0	0	chr21:6445364-6445731

21	43053719	43054053	CBS	0	0	chr21:6445398-6445731
21	43053792	43054053	CBS	0	0	chr21:6445471-6445731
21	43055002	43056169	CBS	0	1	chr21:6446681-6447847
21	43055513	43055971	CBS	0	1	chr21:6447192-6447649
21	43055528	43055940	CBS	0	1	chr21:6447207-6447618
21	43055988	43056169	CBS	0	0	chr21:6447667-6447847
21	43056733	43056957	CBS	0	0	chr21:6448412-6448635
21	43058075	43058323	CBS	0	0	chr21:6449754-6450001
21	43058764	43059038	CBS	0	0	chr21:6450443-6450689
21	43058764	43059038	CBS	0	0	chr21:6450443-6450702
21	43058764	43059011	CBS	0	0	chr21:6450443-6450716
21	43058764	43059011	CBS	0	0	chr21:6450443-6450689
21	43058764	43059011	CBS	0	0	chr21:6450443-6450702
21	43058764	43059024	CBS	0	0	chr21:6450443-6450716
21	43058764	43059024	CBS	0	0	chr21:6450443-6450689
21	43058764	43059024	CBS	0	0	chr21:6450443-6450702
21	43058764	43059038	CBS	0	0	chr21:6450443-6450716
21	43059156	43059373	CBS	0	0	chr21:6450835-6451487
21	43059156	43059809	CBS	0	0	chr21:6450835-6451051
21	43059156	43059809	CBS	0	0	chr21:6450835-6451487
21	43059156	43059373	CBS	0	0	chr21:6450835-6451051
21	43059206	43059373	CBS	0	0	chr21:6450885-6451051
21	43060371	43060590	CBS	0	0	chr21:6452050-6452268
21	43060371	43060590	CBS	0	0	chr21:6452050-6452294
21	43060371	43060616	CBS	0	0	chr21:6452050-6452268
21	43060371	43060616	CBS	0	0	chr21:6452050-6452294
21	43061849	43062465	CBS	0	0	chr21:6453528-6454143
21	43062241	43062465	CBS	0	0	chr21:6453920-6454143

21	43062883	43063318	CBS	0	0	chr21:6454562-6454826
21	43062883	43063148	CBS	0	0	chr21:6454562-6454826
21	43062883	43063148	CBS	0	0	chr21:6454562-6454996
21	43062883	43063318	CBS	0	0	chr21:6454562-6454996
21	43063830	43064297	CBS	0	0	chr21:6455509-6455975
21	43063830	43064297	CBS	0	0	chr21:6455509-6455739
21	43063830	43064061	CBS	0	0	chr21:6455509-6455975
21	43063830	43064061	CBS	0	0	chr21:6455509-6455739
21	43065133	43065342	CBS	0	1	chr21:6456812-6457020
21	43065317	43065591	CBS	0	0	chr21:6456996-6457269
21	43065546	43065765	CBS	0	0	chr21:6457225-6457443
21	43066173	43066447	CBS	0	1	chr21:6457852-6458125
21	43067708	43068030	CBS	0	2	chr21:6459387-6459708
21	43067708	43068030	CBS	0	3	chr21:6459387-6460152
21	43067708	43068474	CBS	0	3	chr21:6459387-6460152
21	43067708	43068474	CBS	0	2	chr21:6459387-6459708
21	43068251	43068685	CBS	1	1	chr21:6459930-6460364
21	43068334	43068685	CBS	1	0	chr21:6460013-6460364
21	43068439	43068685	CBS	1	0	chr21:6460118-6460364
21	43071915	43072271	CBS	0	0	chr21:6463594-6463949
21	43073196	43073578	CBS	0	0	chr21:6464875-6465051
21	43073196	43073578	CBS	0	0	chr21:6464875-6465089
21	43073196	43073578	CBS	0	0	chr21:6464875-6465256
21	43073196	43073373	CBS	0	0	chr21:6464875-6465051
21	43073196	43073373	CBS	0	0	chr21:6464875-6465089
21	43073196	43073373	CBS	0	0	chr21:6464875-6465256
21	43073196	43073411	CBS	0	0	chr21:6464875-6465256
21	43073196	43073411	CBS	0	0	chr21:6464875-6465089

21	43073196	43073411	CBS	0	0	chr21:6464875-6465051
21	43075037	43075839	CBS	0	0	chr21:6466716-6467517
21	43075700	43075934	CBS	0	0	chr21:6467379-6467612
21	43075700	43075934	CBS	0	1	chr21:6467379-6467693
21	43075700	43075934	CBS	0	0	chr21:6467379-6467579
21	43075700	43076015	CBS	0	0	chr21:6467379-6467612
21	43075700	43076015	CBS	0	1	chr21:6467379-6467693
21	43075700	43076015	CBS	0	0	chr21:6467379-6467579
21	43075700	43075901	CBS	0	0	chr21:6467379-6467612
21	43075700	43075901	CBS	0	1	chr21:6467379-6467693
21	43075700	43075901	CBS	0	0	chr21:6467379-6467579
21	43076155	43076358	CBS	0	0	chr21:6467834-6468126
21	43076155	43076448	CBS	0	0	chr21:6467834-6468126
21	43076155	43076448	CBS	0	0	chr21:6467834-6468036
21	43076155	43076358	CBS	0	0	chr21:6467834-6468110
21	43076155	43076448	CBS	0	0	chr21:6467834-6468110
21	43076155	43076432	CBS	0	0	chr21:6467834-6468036
21	43076155	43076432	CBS	0	0	chr21:6467834-6468110
21	43076155	43076358	CBS	0	0	chr21:6467834-6468036
21	43076155	43076432	CBS	0	0	chr21:6467834-6468126
21	43076670	43077013	CBS	0	1	chr21:6468349-6468691
7	117548571	117548893	CFTR	7	6	chr20:25919736-25919428
7	117548571	117548893	CFTR	7	9	chr20:30215031-30214727
16	30188790	30189104	CORO1A	0	0	chr16:29449452-29449765
16	30188790	30189104	CORO1A	0	0	chr16:29449452-29449695
16	30188790	30189104	CORO1A	0	0	chr16:29449452-29449806
16	30188790	30189104	CORO1A	0	0	chr16:29449452-29449807
16	30188790	30189034	CORO1A	0	0	chr16:29449452-29449765

16	30188790	30189034	CORO1A	0	0	chr16:29449452-29449695
16	30188790	30189034	CORO1A	0	0	chr16:29449452-29449806
16	30188790	30189034	CORO1A	0	0	chr16:29449452-29449807
16	30188790	30189145	CORO1A	0	0	chr16:29449452-29449806
16	30188790	30189145	CORO1A	0	0	chr16:29449452-29449695
16	30188790	30189145	CORO1A	0	0	chr16:29449452-29449807
16	30188790	30189146	CORO1A	0	0	chr16:29449452-29449695
16	30188790	30189146	CORO1A	0	0	chr16:29449452-29449806
16	30188790	30189146	CORO1A	0	0	chr16:29449452-29449807
16	30188790	30189145	CORO1A	0	0	chr16:29449452-29449765
16	30188790	30189146	CORO1A	0	0	chr16:29449452-29449765
15	74359453	74359673	CYP11A1	3	10	chr3:86386958-86387172
15	74359453	74359673	CYP11A1	2	9	chr2:75430762-75430983
15	74359453	74359673	CYP11A1	1	10	chr1:82360490-82360272
15	74359453	74359673	CYP11A1	0	9	chr1:163591375-163591594
15	74359453	74359673	CYP11A1	4	10	chr7:139093485-139093700
15	74359453	74359673	CYP11A1	1	10	chr7:75778978-75779198
15	74359453	74359673	CYP11A1	1	10	chr9:98275237-98275018
6	32038195	32038694	CYP21A2	1	8	chr6:32005461-32005960
6	32038246	32038694	CYP21A2	0	6	chr6:32005513-32005960
6	32038257	32038694	CYP21A2	0	6	chr6:32005524-32005960
6	32038270	32038881	CYP21A2	3	10	chr6:32005537-32006150
6	32038295	32038694	CYP21A2	2	5	chr6:32005564-32005960
6	32038295	32038694	CYP21A2	1	9	chr6:32005564-32006150
6	32038295	32038881	CYP21A2	1	9	chr6:32005564-32006150
6	32038295	32038881	CYP21A2	2	5	chr6:32005564-32005960
6	32038300	32038694	CYP21A2	3	9	chr6:32005567-32006150
6	32038300	32038881	CYP21A2	0	5	chr6:32005567-32005960

6	32038300	32038881	CYP21A2	3	9	chr6:32005567-32006150
6	32038300	32038694	CYP21A2	0	5	chr6:32005567-32005960
6	32038312	32038694	CYP21A2	1	3	chr6:32005580-32005960
6	32038345	32038694	CYP21A2	0	2	chr6:32005612-32005960
6	32038346	32038694	CYP21A2	0	2	chr6:32005613-32005960
6	32038513	32038881	CYP21A2	2	4	chr6:32005781-32006150
6	32038652	32038881	CYP21A2	3	4	chr6:32005919-32006150
6	32038652	32039185	CYP21A2	3	4	chr6:32005919-32006150
6	32039024	32039318	CYP21A2	0	9	chr6:32006297-32006582
6	32039040	32039318	CYP21A2	0	8	chr6:32006313-32006582
6	32039144	32039318	CYP21A2	0	0	chr6:32006409-32006582
6	32039286	32039503	CYP21A2	0	4	chr6:32006551-32006818
6	32039286	32039503	CYP21A2	0	1	chr6:32006551-32006767
6	32039286	32039554	CYP21A2	0	4	chr6:32006551-32006818
6	32039286	32039554	CYP21A2	0	1	chr6:32006551-32006767
6	32039286	32039554	CYP21A2	0	1	chr6:32006551-32006791
6	32039286	32039503	CYP21A2	0	1	chr6:32006551-32006791
6	32039286	32039527	CYP21A2	0	1	chr6:32006551-32006791
6	32039286	32039527	CYP21A2	0	1	chr6:32006551-32006767
6	32039286	32039527	CYP21A2	0	4	chr6:32006551-32006818
6	32039476	32039710	CYP21A2	0	10	chr6:32006741-32007306
6	32039476	32039710	CYP21A2	0	3	chr6:32006741-32006974
6	32039476	32040042	CYP21A2	0	3	chr6:32006741-32006981
6	32039476	32040042	CYP21A2	0	10	chr6:32006741-32007306
6	32039476	32040042	CYP21A2	0	3	chr6:32006741-32006974
6	32039476	32039710	CYP21A2	0	3	chr6:32006741-32006981
6	32039476	32039717	CYP21A2	0	3	chr6:32006741-32006981
6	32039476	32039717	CYP21A2	0	3	chr6:32006741-32006974

6	32039476	32039717	CYP21A2	0	10	chr6:32006741-32007306
6	32039515	32039717	CYP21A2	0	3	chr6:32006780-32006940
6	32039515	32039676	CYP21A2	0	3	chr6:32006780-32006940
6	32039515	32039676	CYP21A2	0	3	chr6:32006780-32006981
6	32039515	32039717	CYP21A2	0	3	chr6:32006780-32006981
6	32039679	32039826	CYP21A2	0	4	chr6:32006944-32007090
6	32039679	32039826	CYP21A2	0	6	chr6:32006944-32007169
6	32039679	32039893	CYP21A2	0	6	chr6:32006944-32007157
6	32039679	32039905	CYP21A2	0	6	chr6:32006944-32007157
6	32039679	32039826	CYP21A2	0	6	chr6:32006944-32007158
6	32039679	32039826	CYP21A2	0	6	chr6:32006944-32007157
6	32039679	32039893	CYP21A2	0	4	chr6:32006944-32007090
6	32039679	32039893	CYP21A2	0	6	chr6:32006944-32007169
6	32039679	32039893	CYP21A2	0	6	chr6:32006944-32007158
6	32039679	32039894	CYP21A2	0	4	chr6:32006944-32007090
6	32039679	32039894	CYP21A2	0	6	chr6:32006944-32007169
6	32039679	32039894	CYP21A2	0	6	chr6:32006944-32007158
6	32039679	32039905	CYP21A2	0	6	chr6:32006944-32007169
6	32039679	32039905	CYP21A2	0	4	chr6:32006944-32007090
6	32039679	32039894	CYP21A2	0	6	chr6:32006944-32007157
6	32039679	32039905	CYP21A2	0	6	chr6:32006944-32007158
6	32039935	32040275	CYP21A2	1	3	chr6:32007200-32007540
6	32040336	32040841	CYP21A2	0	2	chr6:32007602-32007919
6	32040336	32040841	CYP21A2	0	3	chr6:32007602-32008106
6	32040336	32040654	CYP21A2	0	2	chr6:32007602-32007919
6	32040336	32040654	CYP21A2	0	3	chr6:32007602-32008106
6	32040598	32040841	CYP21A2	0	1	chr6:32007864-32008106
6	32040799	32041740	CYP21A2	0	9	chr6:32008065-32008977

6	32040799	32041256	CYP21A2	0	9	chr6:32008065-32009005
6	32040799	32041740	CYP21A2	0	9	chr6:32008065-32009005
6	32040799	32041740	CYP21A2	0	2	chr6:32008065-32008521
6	32040799	32041256	CYP21A2	0	2	chr6:32008065-32008521
6	32040799	32041712	CYP21A2	0	2	chr6:32008065-32008521
6	32040799	32041712	CYP21A2	0	9	chr6:32008065-32008977
6	32040799	32041256	CYP21A2	0	9	chr6:32008065-32008977
6	32040799	32041712	CYP21A2	0	9	chr6:32008065-32009005
10	14934310	14934590	DCLRE1C	0	9	chr10:15016839-15017114
10	14934375	14934590	DCLRE1C	0	6	chr10:15016900-15017114
10	14934633	14934845	DCLRE1C	2	5	chr10:15017158-15017365
10	14934653	14934845	DCLRE1C	2	5	chr10:15017178-15017365
10	14939740	14939939	DCLRE1C	0	4	chr10:15021735-15021933
10	14942044	14942505	DCLRE1C	10	9	chr10:15024054-15024524
10	14942044	14942505	DCLRE1C	10	7	chr10:15024054-15024329
10	14942044	14942310	DCLRE1C	10	7	chr10:15024054-15024329
10	14942044	14942310	DCLRE1C	10	9	chr10:15024054-15024524
15	82214647	82214925	EFL1	0	2	chr15:84126669-84126392
15	82214713	82214925	EFL1	0	2	chr15:84126603-84126392
15	82215513	82215733	EFL1	1	9	chr15:84125799-84125580
15	82219582	82219888	EFL1	1	5	chr15:84121738-84121432
15	82225095	82225334	EFL1	2	2	chr15:84116211-84115980
15	82227396	82227642	EFL1	0	9	chr15:84113760-84113517
15	82228964	82229180	EFL1	5	5	chr15:84112191-84111972
15	82233593	82233844	EFL1	0	8	chr15:84109566-84109316
15	82238237	82238591	EFL1	0	7	chr15:84104888-84104535
15	82238238	82238591	EFL1	0	7	chr15:84104887-84104535
15	82238384	82238591	EFL1	0	1	chr15:84104741-84104535

15	82241200	82241473	EFL1	0	7	chr15:84102233-84101961
15	82252621	82252845	EFL1	0	3	chr15:84090360-84090155
15	82252621	82252845	EFL1	0	3	chr15:84090360-84090139
15	82252621	82252829	EFL1	0	3	chr15:84090360-84090155
15	82252621	82252829	EFL1	0	3	chr15:84090360-84090139
15	82259018	82259225	EFL1	5	8	chr15:84084336-84084125
15	82261618	82261867	EFL1	0	6	chr15:84081314-84081070
15	82261659	82261861	EFL1	0	4	chr15:84081273-84081076
22	23573299	23573655	IGLL1	3	8	chr22:25320049-25319697
7	56017164	56017449	PSPH	1	6	chr7:55765013-55764728
7	56017207	56017449	PSPH	1	5	chr7:55764970-55764728
7	66988271	66988569	SBDS	0	10	chr7:72836803-72836507
7	66991067	66991371	SBDS	0	3	chr7:72833997-72833694
7	66993147	66993487	SBDS	3	6	chr7:72831867-72831535
7	66994142	66994412	SBDS	0	5	chr7:72830890-72830621
7	66994142	66994411	SBDS	0	5	chr7:72830890-72830622
7	66994142	66994411	SBDS	0	5	chr7:72830890-72830621
7	66994142	66994412	SBDS	0	5	chr7:72830890-72830622
7	66994150	66994436	SBDS	0	5	chr7:72830882-72830597
7	66994150	66994411	SBDS	0	5	chr7:72830882-72830597
7	66994150	66994436	SBDS	0	5	chr7:72830882-72830622
7	66994150	66994411	SBDS	0	5	chr7:72830882-72830622
7	66995220	66995363	SBDS	0	3	chr7:72829852-72829710
7	66995220	66995363	SBDS	0	8	chr7:72829852-72829437
7	66995220	66995363	SBDS	0	9	chr7:72829852-72829427
7	66995220	66995363	SBDS	0	10	chr7:72829852-72829402
7	66995220	66995636	SBDS	0	3	chr7:72829852-72829710
7	66995220	66995636	SBDS	0	8	chr7:72829852-72829437

7	66995220	66995636	SBDS	0	9	chr7:72829852-72829427
7	66995220	66995636	SBDS	0	10	chr7:72829852-72829402
7	66995220	66995646	SBDS	0	3	chr7:72829852-72829710
7	66995220	66995646	SBDS	0	9	chr7:72829852-72829427
7	66995220	66995646	SBDS	0	10	chr7:72829852-72829402
7	66995220	66995671	SBDS	0	3	chr7:72829852-72829710
7	66995220	66995671	SBDS	0	8	chr7:72829852-72829437
7	66995220	66995671	SBDS	0	9	chr7:72829852-72829427
7	66995220	66995671	SBDS	0	10	chr7:72829852-72829402
7	66995220	66995646	SBDS	0	8	chr7:72829852-72829437
7	66995225	66995657	SBDS	0	9	chr7:72829847-72829416
1	112956769	112957083	SLC16A1	0	8	chr1:112850241-112850554
13	40808368	40808666	SLC25A15	4	5	chr22:16646679-16646504
13	40808368	40808666	SLC25A15	4	6	chr21:10613444-10613269
13	40808368	40808666	SLC25A15	4	9	chr13:24943511-24943685
13	40808368	40808666	SLC25A15	5	9	chrY:26587630-26587454
13	40808368	40808666	SLC25A15	5	6	chr13:19415202-19415377
13	40808368	40808540	SLC25A15	5	9	chrY:26587630-26587454
13	40808368	40808540	SLC25A15	4	6	chr21:10613444-10613269
13	40808368	40808540	SLC25A15	4	5	chr22:16646679-16646504
13	40808368	40808540	SLC25A15	4	9	chr13:24943511-24943685
13	40808368	40808540	SLC25A15	5	6	chr13:19415202-19415377
X	153688653	153688906	SLC6A8	0	9	chr16:32885422-32885170
X	153688653	153688906	SLC6A8	0	10	chr16:33976118-33976370
X	153689397	153689669	SLC6A8	0	7	chr16:33976871-33977134
X	153689397	153689669	SLC6A8	0	9	chr16:32884665-32884402
X	153690305	153690576	SLC6A8	0	8	chr16:32883772-32883502
X	153690305	153690576	SLC6A8	0	8	chr16:33977764-33978034

X	153690305	153690722	SLC6A8	0	8	chr16:32883772-32883502
X	153690305	153690722	SLC6A8	0	8	chr16:33977764-33978034
X	153691234	153691623	SLC6A8	0	10	chr16:32882848-32882461
X	153691234	153691623	SLC6A8	0	10	chr16:33978688-33979075
X	153691293	153691577	SLC6A8	0	7	chr16:32882790-32882507
X	153691293	153691577	SLC6A8	0	7	chr16:33978746-33979029
X	153691391	153691623	SLC6A8	0	7	chr16:32882692-32882461
X	153691391	153691623	SLC6A8	0	7	chr16:33978844-33979075
X	153691905	153692152	SLC6A8	0	7	chr16:33979380-33979759
X	153691905	153692152	SLC6A8	0	3	chr16:33979380-33979626
X	153691905	153692152	SLC6A8	0	3	chr16:32882156-32881910
X	153691905	153692152	SLC6A8	0	4	chr16:33979380-33979651
X	153691905	153692152	SLC6A8	0	4	chr16:32882156-32881885
X	153691905	153692177	SLC6A8	0	8	chr16:32882156-32881777
X	153691905	153692177	SLC6A8	0	7	chr16:33979380-33979759
X	153691905	153692177	SLC6A8	0	3	chr16:32882156-32881910
X	153691905	153692177	SLC6A8	0	4	chr16:33979380-33979651
X	153691905	153692177	SLC6A8	0	4	chr16:32882156-32881885
X	153691905	153692177	SLC6A8	0	3	chr16:33979380-33979626
X	153691905	153692152	SLC6A8	0	8	chr16:32882156-32881777
X	153691905	153692285	SLC6A8	0	8	chr16:32882156-32881777
X	153691905	153692285	SLC6A8	0	7	chr16:33979380-33979759
X	153691905	153692285	SLC6A8	0	3	chr16:33979380-33979626
X	153691905	153692285	SLC6A8	0	3	chr16:32882156-32881910
X	153691905	153692285	SLC6A8	0	4	chr16:33979380-33979651
X	153691905	153692285	SLC6A8	0	4	chr16:32882156-32881885
X	153693257	153693436	SLC6A8	0	6	chr16:33980735-33980908
X	153693257	153693436	SLC6A8	0	6	chr16:32880801-32880628

X	153693422	153693656	SLC6A8	1	7	chr16:33980895-33981127
X	153693422	153693656	SLC6A8	1	8	chr16:32880641-32880409
X	153693835	153694087	SLC6A8	0	9	chr16:33981308-33981514
X	153693835	153694042	SLC6A8	0	8	chr16:32880228-32880022
X	153693835	153694042	SLC6A8	0	9	chr16:33981308-33981514
X	153693835	153694087	SLC6A8	0	8	chr16:32880228-32880022
X	153693835	153694703	SLC6A8	0	9	chr16:33981308-33981514
X	153693835	153694703	SLC6A8	0	8	chr16:32880228-32880022
X	153694060	153694337	SLC6A8	2	9	chr16:32880001-32879727
X	153694060	153694337	SLC6A8	2	10	chr16:33981535-33981809
X	153694067	153694234	SLC6A8	0	7	chr16:32879996-32879830
X	153694067	153694234	SLC6A8	0	7	chr16:33981540-33981706
X	153694134	153694337	SLC6A8	0	5	chr16:32879929-32879727
X	153694134	153694337	SLC6A8	0	6	chr16:33981607-33981809
X	153694274	153694490	SLC6A8	0	7	chr16:33981747-33981988
X	153694274	153694490	SLC6A8	0	7	chr16:33981747-33981962
X	153694274	153694490	SLC6A8	0	7	chr16:32879789-32879574
X	153694274	153694490	SLC6A8	0	7	chr16:32879789-32879548
X	153694274	153694516	SLC6A8	0	7	chr16:33981747-33981962
X	153694274	153694516	SLC6A8	0	7	chr16:32879789-32879574
X	153694274	153694516	SLC6A8	0	7	chr16:33981747-33981988
X	153694274	153694516	SLC6A8	0	7	chr16:32879789-32879548
X	153694463	153694703	SLC6A8	0	6	chr16:32879600-32879418
X	153694463	153694646	SLC6A8	4	10	chr16:32879600-32879368
X	153694463	153694646	SLC6A8	0	6	chr16:32879600-32879418
X	153694463	153694646	SLC6A8	0	7	chr16:33981936-33982118
X	153694463	153694703	SLC6A8	0	7	chr16:33981936-33982118
X	153694463	153694703	SLC6A8	4	10	chr16:32879600-32879368

5	70924960	70925254	SMN1	0	0	chr5:70049543-70049836
5	70924986	70925254	SMN1	0	0	chr5:70049569-70049836
5	70925005	70925254	SMN1	0	0	chr5:70049588-70049836
5	70925025	70925254	SMN1	0	0	chr5:70049608-70049836
5	70925034	70925254	SMN1	0	0	chr5:70049617-70049836
5	70938769	70938980	SMN1	0	0	chr5:70063346-70063556
5	70941319	70941578	SMN1	0	0	chr5:70065896-70066154
5	70942288	70942628	SMN1	0	0	chr5:70066865-70067204
5	70942402	70942628	SMN1	0	0	chr5:70066979-70067204
5	70942438	70942628	SMN1	0	0	chr5:70067015-70067204
5	70942448	70942628	SMN1	0	0	chr5:70067025-70067204
5	70942648	70942940	SMN1	0	0	chr5:70067225-70067516
5	70944379	70944635	SMN1	0	0	chr5:70068957-70069212
5	70944588	70944823	SMN1	0	0	chr5:70069166-70069400
5	70945996	70946149	SMN1	0	0	chr5:70070572-70070724
5	70945996	70946348	SMN1	0	0	chr5:70070572-70070724
5	70945996	70946348	SMN1	0	0	chr5:70070572-70070919
5	70945996	70946149	SMN1	0	0	chr5:70070572-70070821
5	70945996	70946149	SMN1	0	0	chr5:70070572-70070923
5	70945996	70946344	SMN1	0	0	chr5:70070572-70070821
5	70945996	70946149	SMN1	0	0	chr5:70070572-70070919
5	70945996	70946344	SMN1	0	0	chr5:70070572-70070923
5	70945996	70946344	SMN1	0	0	chr5:70070572-70070724
5	70945996	70946344	SMN1	0	0	chr5:70070572-70070919
5	70945996	70946348	SMN1	0	0	chr5:70070572-70070923
5	70945996	70946246	SMN1	0	0	chr5:70070572-70070821
5	70945996	70946246	SMN1	0	0	chr5:70070572-70070923
5	70945996	70946246	SMN1	0	0	chr5:70070572-70070724

5	70945996	70946348	SMN1	0	0	chr5:70070572-70070821
5	70945996	70946246	SMN1	0	0	chr5:70070572-70070919
5	70946019	70946246	SMN1	0	0	chr5:70070595-70070821
5	70946637	70946827	SMN1	0	0	chr5:70071213-70071460
5	70946637	70946827	SMN1	0	0	chr5:70071213-70071402
5	70946637	70946885	SMN1	0	0	chr5:70071213-70071460
5	70946637	70946885	SMN1	0	0	chr5:70071213-70071402
5	70951871	70952637	SMN1	0	4	chr5:70076452-70077217
5	70951871	70952637	SMN1	0	2	chr5:70076452-70076644
5	70951871	70952637	SMN1	0	4	chr5:70076452-70076997
5	70951871	70952064	SMN1	0	2	chr5:70076452-70076656
5	70951871	70952637	SMN1	0	2	chr5:70076452-70076656
5	70951871	70952076	SMN1	0	2	chr5:70076452-70076656
5	70951871	70952076	SMN1	0	4	chr5:70076452-70077217
5	70951871	70952076	SMN1	0	2	chr5:70076452-70076644
5	70951871	70952076	SMN1	0	4	chr5:70076452-70076997
5	70951871	70952417	SMN1	0	4	chr5:70076452-70076997
5	70951871	70952417	SMN1	0	2	chr5:70076452-70076644
5	70951871	70952064	SMN1	0	4	chr5:70076452-70077217
5	70951871	70952417	SMN1	0	4	chr5:70076452-70077217
5	70951871	70952064	SMN1	0	2	chr5:70076452-70076644
5	70951871	70952417	SMN1	0	2	chr5:70076452-70076656
5	70951871	70952064	SMN1	0	4	chr5:70076452-70076997
5	70952369	70953110	SMN1	0	1	chr5:70076950-70077690
5	70952369	70953110	SMN1	0	1	chr5:70076950-70077662
5	70952369	70953086	SMN1	0	0	chr5:70076950-70077230
5	70952369	70953110	SMN1	0	1	chr5:70076950-70077666
5	70952369	70952650	SMN1	0	0	chr5:70076950-70077230

5	70952369	70952650	SMN1	0	1	chr5:70076950-70077690
5	70952369	70953110	SMN1	0	0	chr5:70076950-70077230
5	70952369	70952650	SMN1	0	1	chr5:70076950-70077666
5	70952369	70952650	SMN1	0	1	chr5:70076950-70077662
5	70952369	70953082	SMN1	0	1	chr5:70076950-70077662
5	70952369	70953082	SMN1	0	1	chr5:70076950-70077666
5	70952369	70953082	SMN1	0	1	chr5:70076950-70077690
5	70952369	70953086	SMN1	0	1	chr5:70076950-70077690
5	70952369	70953086	SMN1	0	1	chr5:70076950-70077666
5	70952369	70953086	SMN1	0	1	chr5:70076950-70077662
5	70952369	70953082	SMN1	0	0	chr5:70076950-70077230
5	70953112	70954012	SMN1	0	3	chr5:70077693-70078592
5	70049542	70049836	SMN2	0	0	chr5:70924961-70925254
5	70049568	70049836	SMN2	0	0	chr5:70924987-70925254
5	70049584	70049836	SMN2	0	0	chr5:70925003-70925254
5	70049587	70049836	SMN2	0	0	chr5:70925006-70925254
5	70049594	70049836	SMN2	0	0	chr5:70925013-70925254
5	70049616	70049836	SMN2	0	0	chr5:70925035-70925254
5	70063341	70063556	SMN2	0	0	chr5:70938766-70938980
5	70063345	70063556	SMN2	0	0	chr5:70938770-70938980
5	70065895	70066154	SMN2	0	0	chr5:70941320-70941578
5	70066864	70067204	SMN2	0	0	chr5:70942289-70942628
5	70067014	70067204	SMN2	0	0	chr5:70942439-70942628
5	70067024	70067204	SMN2	0	0	chr5:70942449-70942628
5	70067029	70067204	SMN2	0	0	chr5:70942454-70942628
5	70067209	70067516	SMN2	0	0	chr5:70942634-70942940
5	70067224	70067516	SMN2	0	0	chr5:70942649-70942940
5	70068956	70069212	SMN2	0	0	chr5:70944380-70944635

5	70069163	70069400	SMN2	0	0	chr5:70944587-70944823
5	70069165	70069400	SMN2	0	0	chr5:70944589-70944823
5	70070571	70070919	SMN2	0	0	chr5:70945997-70946250
5	70070571	70070919	SMN2	0	0	chr5:70945997-70946149
5	70070571	70070724	SMN2	0	0	chr5:70945997-70946344
5	70070571	70070724	SMN2	0	0	chr5:70945997-70946248
5	70070571	70070919	SMN2	0	0	chr5:70945997-70946246
5	70070571	70070919	SMN2	0	0	chr5:70945997-70946344
5	70070571	70070724	SMN2	0	0	chr5:70945997-70946149
5	70070571	70070823	SMN2	0	0	chr5:70945997-70946248
5	70070571	70070823	SMN2	0	0	chr5:70945997-70946344
5	70070571	70070823	SMN2	0	0	chr5:70945997-70946149
5	70070571	70070823	SMN2	0	0	chr5:70945997-70946250
5	70070571	70070823	SMN2	0	0	chr5:70945997-70946246
5	70070571	70070919	SMN2	0	0	chr5:70945997-70946248
5	70070571	70070821	SMN2	0	0	chr5:70945997-70946248
5	70070571	70070821	SMN2	0	0	chr5:70945997-70946344
5	70070571	70070821	SMN2	0	0	chr5:70945997-70946149
5	70070571	70070821	SMN2	0	0	chr5:70945997-70946250
5	70070571	70070821	SMN2	0	0	chr5:70945997-70946246
5	70070571	70070825	SMN2	0	0	chr5:70945997-70946246
5	70070571	70070724	SMN2	0	0	chr5:70945997-70946250
5	70070571	70070724	SMN2	0	0	chr5:70945997-70946246
5	70070571	70070825	SMN2	0	0	chr5:70945997-70946248
5	70070571	70070825	SMN2	0	0	chr5:70945997-70946344
5	70070571	70070825	SMN2	0	0	chr5:70945997-70946250
5	70070571	70070825	SMN2	0	0	chr5:70945997-70946149
5	70070594	70070821	SMN2	0	0	chr5:70946020-70946246

5	70071101	70071252	SMN2	0	10	chr2:2922897-2922748
5	70071101	70071252	SMN2	1	10	chr2:100579768-100579918
5	70071101	70071252	SMN2	1	10	chr7:116514450-116514300
5	70071101	70071252	SMN2	0	10	chr6:107041233-107041084
5	70071101	70071252	SMN2	1	8	chr6:161320892-161320742
5	70071101	70071252	SMN2	1	10	chr1:174196233-174196383
5	70071101	70071252	SMN2	1	9	chr4:164978714-164978864
5	70071101	70071252	SMN2	1	10	chr4:138228767-138228617
5	70071101	70071252	SMN2	1	9	chr4:36594102-36593952
5	70071101	70071252	SMN2	1	10	chr16:57063748-57063898
5	70071101	70071252	SMN2	0	10	chr10:74087357-74087506
5	70071101	70071252	SMN2	1	10	chr16:74595545-74595397
5	70071101	70071252	SMN2	1	9	chr19:21469174-21469324
5	70071101	70071252	SMN2	1	10	chr19:31940436-31940286
5	70071101	70071252	SMN2	1	10	chr19:43618044-43618194
5	70071101	70071252	SMN2	4	9	chr12:100066812-100066667
5	70071101	70071252	SMN2	1	9	chr12:118317310-118317458
5	70071101	70071252	SMN2	0	10	chr12:13419883-13419733
5	70071101	70071252	SMN2	0	10	chr12:31454828-31454978
5	70071101	70071252	SMN2	0	10	chr12:86042876-86042726
5	70071101	70071252	SMN2	1	10	chr12:25158237-25158087
5	70071101	70071252	SMN2	1	10	chr12:46931494-46931344
5	70071101	70071252	SMN2	1	10	chr12:77015749-77015899
5	70071101	70071252	SMN2	1	10	chr12:108444537-108444387
5	70071101	70071252	SMN2	0	10	chr3:10812968-10813118
5	70071101	70071252	SMN2	1	10	chr5:31257174-31257321
5	70071101	70071252	SMN2	0	1	chr5:70946527-70946677

5	70071101	70071252	SMN2	0	10	chr5:176378901-176378751
5	70071101	70071252	SMN2	1	9	chrX:84094652-84094503
5	70071101	70071252	SMN2	5	10	chrX:30832980-30833125
5	70071101	70071252	SMN2	0	10	chr13:21772924-21772774
5	70071101	70071252	SMN2	0	10	chrX:49801611-49801461
5	70071101	70071252	SMN2	1	9	chrX:51585737-51585887
5	70071101	70071252	SMN2	6	10	chr11:97084300-97084156
5	70071101	70071252	SMN2	4	8	chr8:87005378-87005233
5	70071101	70071252	SMN2	1	10	chr8:125685538-125685688
5	70071101	70071252	SMN2	0	9	chr17:74292839-74292988
5	70071101	70071252	SMN2	1	10	chr17:29349731-29349881
5	70071101	70071252	SMN2	1	10	chr20:14485710-14485559
5	70071101	70071252	SMN2	0	10	chr14:96287262-96287113
5	70071101	70071252	SMN2	1	9	chr15:75091635-75091485
5	70071101	70071252	SMN2	1	10	chr15:85575700-85575550
5	70071212	70071402	SMN2	0	0	chr5:70946638-70946885
5	70071212	70071402	SMN2	0	0	chr5:70946638-70946827
5	70071212	70071460	SMN2	0	0	chr5:70946638-70946885
5	70071212	70071460	SMN2	0	0	chr5:70946638-70946827
5	70076451	70076997	SMN2	0	4	chr5:70951872-70952417
5	70076451	70076997	SMN2	0	3	chr5:70951872-70952196
5	70076451	70076997	SMN2	0	2	chr5:70951872-70952064
5	70076451	70076997	SMN2	0	2	chr5:70951872-70952076
5	70076451	70076656	SMN2	0	2	chr5:70951872-70952064
5	70076451	70076656	SMN2	0	3	chr5:70951872-70952196
5	70076451	70076656	SMN2	0	4	chr5:70951872-70952417
5	70076451	70076656	SMN2	0	2	chr5:70951872-70952076
5	70076451	70076776	SMN2	0	2	chr5:70951872-70952076

5	70076451	70076644	SMN2	0	2	chr5:70951872-70952064
5	70076451	70076644	SMN2	0	3	chr5:70951872-70952196
5	70076451	70076644	SMN2	0	4	chr5:70951872-70952417
5	70076451	70076644	SMN2	0	2	chr5:70951872-70952076
5	70076451	70076776	SMN2	0	4	chr5:70951872-70952417
5	70076451	70076776	SMN2	0	3	chr5:70951872-70952196
5	70076451	70076776	SMN2	0	2	chr5:70951872-70952064
5	70076949	70077321	SMN2	0	1	chr5:70952370-70952741
5	70076949	70077321	SMN2	0	1	chr5:70952370-70953082
5	70076949	70077321	SMN2	0	1	chr5:70952370-70953086
5	70076949	70077321	SMN2	0	0	chr5:70952370-70952650
5	70076949	70077230	SMN2	0	1	chr5:70952370-70953082
5	70076949	70077230	SMN2	0	1	chr5:70952370-70953086
5	70076949	70077230	SMN2	0	1	chr5:70952370-70952741
5	70076949	70077230	SMN2	0	0	chr5:70952370-70952650
5	70076949	70077666	SMN2	0	0	chr5:70952370-70952650
5	70076949	70077662	SMN2	0	1	chr5:70952370-70953082
5	70076949	70077662	SMN2	0	1	chr5:70952370-70953086
5	70076949	70077662	SMN2	0	1	chr5:70952370-70952741
5	70076949	70077662	SMN2	0	0	chr5:70952370-70952650
5	70076949	70077666	SMN2	0	1	chr5:70952370-70952741
5	70076949	70077666	SMN2	0	1	chr5:70952370-70953082
5	70076949	70077666	SMN2	0	1	chr5:70952370-70953086
5	70077692	70078592	SMN2	0	3	chr5:70953113-70954012
17	42218653	42218948	STAT5B	0	3	chr17:42300939-42300645
17	42219242	42219533	STAT5B	0	1	chr17:42300350-42300060
17	42219642	42219912	STAT5B	0	6	chr1742299950-42299681

Supplementary Table 2. Genes with BLAST+ hits or GEM scores ≤ 0.5 which were then used for simulation analyses.

Chromosome	Start	End	Gene Name
X	153724856	153744755	ABCD1
1	75724347	75787575	ACADM
12	120725774	120740008	ACADS
10	123008979	123058311	ACADSB
17	7217125	7225266	ACADVL
11	108121516	108147776	ACAT1
20	44619522	44652233	ADA
10	74151185	74709303	ADK
20	34280268	34311802	AHCY
1	33007940	33080996	AK2
5	126531200	126595362	ALDH7A1
6	131573144	131584332	ARG1
7	99374249	99394816	ARPC1B
22	50622754	50628173	ARSA
7	66075798	66093558	ASL
9	130444929	130501274	ASS1
11	108222484	108369102	ATM
19	41397808	41425002	BCKDHA
6	80106647	80346270	BCKDHB
16	31106107	31112791	BCKDK
14	99169287	99272197	BCL11B
10	96191702	96271587	BLNK
3	15601341	15647640	BTD
X	101349447	101390796	BTK
16	67644988	67657569	CARMIL2
21	43053191	43076943	CBS

1	167430640	167518610	CD247
11	118338954	118342744	CD3D
11	118304545	118316175	CD3E
11	118344344	118355161	CD3G
19	41877120	41881372	CD79A
7	117465784	117715971	CFTR
8	60678740	60868028	CHD7
16	30182827	30189076	CORO1A
2	210477682	210679107	CPS1
11	68754620	68844410	CPT1A
1	53196792	53214197	CPT2
15	74337759	74367646	CYP11A1
10	102830531	102837472	CYP17A1
6	32038265	32041670	CYP21A2
1	100186919	100249834	DBT
10	14897359	14954432	DCLRE1C
X	154762742	154777689	DKC1
7	107891162	107931730	DLD
X	31097677	33339441	DMD
20	32762385	32809356	DNMT3B
5	169637247	170083382	DOCK2
9	214854	465259	DOCK8
15	82130233	82262734	EFL1
15	76215353	76311472	ETFA
19	51345169	51366418	ETFB
4	158672125	158709623	ETFDH
8	28600469	28755599	EXTL3
15	80152490	80186946	FAH

17	28506243	28538896	FOXN1
17	80101556	80119881	GAA
1	23795599	23800781	GALE
17	75751594	75765711	GALK1
3	128479427	128493185	GATA2
15	45361124	45402327	GATM
19	12891160	12914207	GCDH
2	26190635	26244672	HADHA
2	26243170	26290465	HADHB
21	36750888	36990236	HLCS
1	23801885	23838620	HMGCL
1	119747996	119768905	HMGCS2
12	121839527	121863596	HPD
4	174490175	174523154	HPGD
4	986997	1004564	IDUA
22	23573125	23580302	IGLL1
8	42271302	42332653	IKBKB
7	50304068	50405101	IKZF1
5	35852695	35879603	IL7R
15	40405485	40435947	IVD
19	17824780	17848071	JAK3
16	28984826	28990783	LAT
1	32251239	32286165	LCK
13	108207439	108218368	LIG4
6	69672757	69867236	LMBRD1
9	128882112	128918039	LRRC8A
1	89524836	89597864	LRRC8B
1	89633072	89769903	LRRC8C

1	89821014	89936611	LRRC8D
19	7888505	7902021	LRRC8E
18	13882044	13915707	MC2R
3	183015218	183116075	MCCC1
5	71587288	71658706	MCCC2
4	145599042	145660035	MMAA
12	109553715	109573580	MMAB
1	45500300	45513382	MMACHC
2	149569637	149587778	MMADHC
6	49430360	49463253	MMUT
1	11785723	11806920	MTHFR
1	236795292	236921278	MTR
5	36192589	36242279	NADK2
17	44004546	44009063	NAGS
8	89933336	90003228	NBN
2	219075317	219160865	NHEJ1
18	23506184	23586506	NPC1
14	74476192	74494177	NPC2
7	44379119	44490658	NUDCD3
X	38352586	38421446	OTC
12	102836889	102958410	PAH
20	21705659	21718486	PAX1
10	70882280	70888565	PCBD1
13	100089015	100530437	PCCA
3	136250340	136337896	PCCB
6	83161150	83193936	PGM3
1	9651732	9729114	PIK3CD
5	68215756	68301821	PIK3R1

14	20468954	20477094	PNP
7	75899200	75986855	POR
8	47773108	47960183	PRKDC
10	71816298	71851325	PSAP
9	78297125	78330093	PSAT1
7	56011051	56051604	PSPH
1	198638457	198757476	PTPRC
11	112226367	112269955	PTS
4	17460261	17512206	QDPR
22	37225270	37244448	RAC2
11	36510709	36593156	RAG1
11	36575574	36598279	RAG2
8	144511288	144517845	RECQL4
7	66987680	66995587	SBDS
1	112911847	112957013	SLC16A1
5	132369752	132395614	SLC22A5
7	96120220	96322147	SLC25A13
13	40789412	40812460	SLC25A15
3	48856926	48898904	SLC25A20
17	28394642	28407197	SLC46A1
20	760080	776015	SLC52A3
X	153687926	153696588	SLC6A8
2	216412414	216483053	SMARCAL1
5	70925030	70953942	SMN1
5	70049612	70078522	SMN2
17	42199168	42276707	STAT5B
11	3854527	4093210	STIM1
16	71565660	71577092	TAT

22	19756703	19783593	TBX1
2	46916157	47076137	TTC7A
17	28546707	28552668	UNC119
X	48676596	48691427	WAS
2	97713560	97739862	ZAP70
6	109462594	109483237	ZBTB24

Supplementary Table 3. Mapping accuracy of 150bp reads in simulated NBS genes by population

MQ Threshold	Population	% Correct	% Incorrect	% Unmapped
10	GIH	99.44	0.0002	0.56
	CLM	99.44	0.0004	0.56
	CHS	99.44	0.0003	0.56
	FIN	99.44	0.0003	0.56
	GWD	99.44	0.0003	0.56
20	GIH	99.38	0.0002	0.62
	CLM	99.38	0.0003	0.62
	CHS	99.38	0.0003	0.62
	FIN	99.38	0.0002	0.62
	GWD	99.38	0.0002	0.62

Supplementary Table 4. Mapping accuracy of reads in simulated NBS genes by read length across all populations

MQ Threshold	Read Length	% Correct	% Incorrect	% Unmapped
10	70	99.17	0.0010	0.83
	100	99.32	0.0004	0.68
	150	99.44	0.0003	0.56
	250	99.54	0.0003	0.46
20	70	99.02	0.0006	0.98
	100	99.22	0.0003	0.78
	150	99.38	0.0002	0.62
	250	99.51	0.0002	0.48

Supplementary Table 5. Simulated NBS genes with low depth (<20X) regions with MQ≥10 filter in short read lengths.

Gene	70bp read length	100bp read length	150bp read length	250bp read length
ACADM	LOW DEPTH	OK	OK	OK
ACADS	LOW DEPTH	OK	OK	OK
ADK	LOW DEPTH	LOW DEPTH	OK	OK
AK2	LOW DEPTH	LOW DEPTH	LOW DEPTH	OK
ATM	LOW DEPTH	LOW DEPTH	LOW DEPTH	OK
BCKDHB	LOW DEPTH	LOW DEPTH	OK	OK
CBS	LOW DEPTH	LOW DEPTH	LOW DEPTH	LOW DEPTH
CHD7	LOW DEPTH	LOW DEPTH	OK	OK
CD247	LOW DEPTH	OK	OK	OK
CFTR	LOW DEPTH	LOW DEPTH	LOW DEPTH	LOW DEPTH
CORO1A	LOW DEPTH	LOW DEPTH	LOW DEPTH	LOW DEPTH
CPS1	LOW DEPTH	LOW DEPTH	OK	OK
CYP21A2	LOW DEPTH	LOW DEPTH	OK	OK

DBT	LOW DEPTH	OK	OK	OK
DCLRE1C	LOW DEPTH	OK	OK	OK
DMD	LOW DEPTH	LOW DEPTH	LOW DEPTH	LOW DEPTH
DKC1	LOW DEPTH	OK	OK	OK
DOCK2	LOW DEPTH	LOW DEPTH	OK	OK
DOCK8	LOW DEPTH	LOW DEPTH	LOW DEPTH	OK
EFL1	LOW DEPTH	LOW DEPTH	LOW DEPTH	LOW DEPTH
ETFA	LOW DEPTH	LOW DEPTH	OK	OK
ETFB	LOW DEPTH	OK	OK	OK
IKBKB	LOW DEPTH	OK	OK	OK
LMBRD1	LOW DEPTH	OK	OK	OK
LRRC8B	LOW DEPTH	LOW DEPTH	LOW DEPTH	OK
LRRC8C	LOW DEPTH	LOW DEPTH	LOW DEPTH	OK
MCCC2	LOW DEPTH	LOW DEPTH	LOW DEPTH	OK
NADK2	LOW DEPTH	OK	OK	OK
NUDCD3	LOW DEPTH	OK	OK	OK
PAH	LOW DEPTH	OK	OK	OK
PCCA	LOW DEPTH	LOW DEPTH	OK	OK
PIK3CD	LOW DEPTH	OK	OK	OK
PRKDC	LOW DEPTH	OK	OK	OK
QDPR	LOW DEPTH	OK	OK	OK
RAG1	LOW DEPTH	LOW DEPTH	LOW DEPTH	LOW DEPTH
SBDS	LOW DEPTH	OK	OK	OK
SLC25A13	LOW DEPTH	OK	OK	OK
SMARCAL1	LOW DEPTH	LOW DEPTH	OK	OK
SMN1	LOW DEPTH	LOW DEPTH	LOW DEPTH	LOW DEPTH
SMN2	LOW DEPTH	LOW DEPTH	LOW DEPTH	LOW DEPTH
STAT5B	LOW DEPTH	LOW DEPTH	LOW DEPTH	OK

STIM1	LOW DEPTH	LOW DEPTH	OK	OK
ZNF518A	LOW DEPTH	OK	OK	OK

Supplementary Table 6. Simulated NBS genes with low depth (<20X) exon regions with MQ≥10 filter in short read lengths.

Gene	70bp read length	100bp read length	150bp read length	250bp read length
CBS	LOW DEPTH	LOW DEPTH	LOW DEPTH	LOW DEPTH
CD247	LOW DEPTH	OK	OK	OK
CORO1A	LOW DEPTH	LOW DEPTH	LOW DEPTH	LOW DEPTH
CYP21A2	LOW DEPTH	LOW DEPTH	OK	OK
DKC1	LOW DEPTH	OK	OK	OK
DOCK8	LOW DEPTH	LOW DEPTH	LOW DEPTH	OK
ETFA	LOW DEPTH	OK	OK	OK
SMN1	LOW DEPTH	LOW DEPTH	LOW DEPTH	LOW DEPTH
SMN2	LOW DEPTH	LOW DEPTH	LOW DEPTH	LOW DEPTH
STAT5B	LOW DEPTH	LOW DEPTH	LOW DEPTH	OK

Supplementary Table 7. Low coverage regions with 70bp read lengths

Chromosome	Start position	End Position	Gene
chr1	9675191	9675198	PIK3CD
chr1	9675314	9675347	PIK3CD
chr1	33051118	33051148	AK2
chr1	33051222	33051864	AK2
chr1	33052092	33052364	AK2
chr1	33052619	33052672	AK2
chr1	33052761	33052781	AK2
chr1	33053065	33053435	AK2
chr1	75782101	75782119	ACADM

chr1	75782218	75782252	ACADM
chr1	75782959	75782973	ACADM
chr1	89541491	89541671	LRRC8B
chr1	89560563	89560885	LRRC8B
chr1	89560939	89561834	LRRC8B
chr1	89704842	89705173	LRRC8C
chr1	89705239	89705260	LRRC8C
chr1	89747094	89747973	LRRC8C
chr1	89748051	89748099	LRRC8C
chr1	89748203	89749887	LRRC8C
chr1	89749962	89751920	LRRC8C
chr1	89752008	89752033	LRRC8C
chr1	89752126	89752614	LRRC8C
chr1	100210327	100210346	DBT
chr1	167435134	167435177	CD247
chr10	14940525	14940560	DCLRE1C
chr10	14941707	14941771	DCLRE1C
chr10	14941839	14941892	DCLRE1C
chr10	74217666	74217698	ADK
chr10	74217765	74217975	ADK
chr10	74219032	74219081	ADK
chr10	74219143	74219185	ADK
chr10	74219245	74219311	ADK
chr10	74219362	74219421	ADK
chr10	74219488	74219508	ADK
chr10	74219612	74219846	ADK
chr10	74219914	74220003	ADK
chr10	74220088	74220106	ADK

chr10	74220339	74220562	ADK
chr10	74220943	74221416	ADK
chr10	74221502	74221529	ADK
chr10	74221598	74221877	ADK
chr10	74222196	74222526	ADK
chr10	74222626	74222651	ADK
chr10	74356059	74356262	ADK
chr10	74569323	74569368	ADK
chr10	74569884	74570163	ADK
chr10	74570518	74570555	ADK
chr10	74570641	74570686	ADK
chr10	74570783	74570804	ADK
chr10	74571143	74571427	ADK
chr10	74571696	74571711	ADK
chr10	74572017	74572187	ADK
chr10	74572259	74572288	ADK
chr10	74573076	74573140	ADK
chr10	74573227	74573237	ADK
chr10	96194832	96195006	ZNF518A
chr11	3897704	3897743	STIM1
chr11	3898154	3898319	STIM1
chr11	3898381	3898430	STIM1
chr11	3898490	3898961	STIM1
chr11	3899153	3899202	STIM1
chr11	3899272	3899351	STIM1
chr11	3899402	3899981	STIM1
chr11	4001013	4001046	STIM1
chr11	4001127	4001186	STIM1

chr11	4001248	4001312	STIM1
chr11	4001703	4002269	STIM1
chr11	4002490	4002770	STIM1
chr11	4002843	4002875	STIM1
chr11	4003013	4003609	STIM1
chr11	4003800	4003830	STIM1
chr11	4004210	4004230	STIM1
chr11	4004301	4004819	STIM1
chr11	4018225	4018407	STIM1
chr11	4033009	4033241	STIM1
chr11	36518730	36518763	RAG1
chr11	36551764	36557494	RAG1
chr11	108261483	108261528	ATM
chr11	108261601	108261669	ATM
chr11	108261730	108261782	ATM
chr11	108262528	108262989	ATM
chr11	108263139	108263969	ATM
chr11	108264025	108264112	ATM
chr11	108264497	108265193	ATM
chr11	108265247	108265302	ATM
chr11	108265375	108265448	ATM
chr11	108265514	108265980	ATM
chr11	108266066	108266105	ATM
chr11	108357503	108357528	ATM
chr11	108357795	108357864	ATM
chr11	108357927	108357994	ATM
chr11	108358067	108358102	ATM
chr11	108358166	108358559	ATM

chr11	108358617	108358680	ATM
chr11	108358998	108359368	ATM
chr11	108359428	108359476	ATM
chr11	108359939	108360569	ATM
chr11	108360660	108361421	ATM
chr11	108361490	108361557	ATM
chr11	108361847	108362416	ATM
chr11	108362479	108362767	ATM
chr12	102859432	102859464	PAH
chr12	102859583	102859592	PAH
chr12	102899636	102899827	PAH
chr12	120731697	120731896	ACADS
chr13	100264022	100264091	PCCA
chr13	100283776	100283821	PCCA
chr13	100319022	100319051	PCCA
chr13	100319137	100319537	PCCA
chr13	100320281	100320319	PCCA
chr13	100320411	100320476	PCCA
chr13	100320554	100320606	PCCA
chr15	76230277	76230439	ETFA
chr15	76257497	76257825	ETFA
chr15	82170245	82170297	EFL1
chr15	82193503	82193532	EFL1
chr15	82193609	82193899	EFL1
chr15	82201738	82202008	EFL1
chr15	82214678	82214684	EFL1
chr15	82220519	82220535	EFL1
chr15	82220645	82220672	EFL1

chr15	82234578	82234636	EFL1
chr15	82242292	82243781	EFL1
chr15	82250093	82250593	EFL1
chr16	30188651	30189075	CORO1A
chr17	42218722	42218732	STAT5B
chr17	42218823	42218886	STAT5B
chr17	42219301	42219765	STAT5B
chr17	42221019	42221069	STAT5B
chr17	42264352	42264392	STAT5B
chr17	42264495	42264519	STAT5B
chr17	42264658	42265185	STAT5B
chr19	51353495	51353560	ETFB
chr2	210507485	210507755	CPS1
chr2	210507833	210507857	CPS1
chr2	210508161	210508219	CPS1
chr2	210508283	210508477	CPS1
chr2	210508532	210508538	CPS1
chr2	210509164	210509191	CPS1
chr2	210509292	210509320	CPS1
chr2	210509394	210509433	CPS1
chr2	210510280	210510310	CPS1
chr2	210510783	210510821	CPS1
chr2	210639384	210639584	CPS1
chr2	210665804	210665887	CPS1
chr2	210665941	210666023	CPS1
chr2	210666074	210666503	CPS1
chr2	216458846	216458906	SMARCAL1
chr2	216458965	216459023	SMARCAL1

chr2	216459564	216459885	SMARCAL1
chr2	216460637	216460668	SMARCAL1
chr21	43053191	43055733	CBS
chr21	43055798	43056614	CBS
chr21	43056680	43057288	CBS
chr21	43057362	43057400	CBS
chr21	43057473	43059973	CBS
chr21	43060035	43061748	CBS
chr21	43061813	43063630	CBS
chr21	43063694	43065208	CBS
chr21	43065271	43066102	CBS
chr21	43066271	43066797	CBS
chr21	43067042	43067309	CBS
chr21	43067376	43067747	CBS
chr21	43067826	43067878	CBS
chr21	43067957	43068250	CBS
chr21	43068317	43068607	CBS
chr21	43068768	43070072	CBS
chr21	43070136	43075920	CBS
chr21	43075983	43076798	CBS
chr21	43076863	43076937	CBS
chr4	17469874	17469878	QDPR
chr5	36234156	36234335	NADK2
chr5	70049612	70052171	SMN2
chr5	70052223	70060226	SMN2
chr5	70060291	70068348	SMN2
chr5	70068400	70068746	SMN2
chr5	70068811	70071150	SMN2

chr5	70071214	70072857	SMN2
chr5	70072922	70074592	SMN2
chr5	70074667	70074722	SMN2
chr5	70074814	70074861	SMN2
chr5	70074973	70075011	SMN2
chr5	70075101	70075484	SMN2
chr5	70075737	70075762	SMN2
chr5	70075851	70075907	SMN2
chr5	70076104	70076416	SMN2
chr5	70076707	70076756	SMN2
chr5	70076826	70077222	SMN2
chr5	70077286	70077775	SMN2
chr5	70077899	70078521	SMN2
chr5	70925030	70935648	SMN1
chr5	70935715	70944169	SMN1
chr5	70944236	70946576	SMN1
chr5	70946639	70947121	SMN1
chr5	70947181	70948283	SMN1
chr5	70948348	70950016	SMN1
chr5	70950091	70950144	SMN1
chr5	70950240	70950284	SMN1
chr5	70950397	70950436	SMN1
chr5	70950525	70950910	SMN1
chr5	70951153	70951211	SMN1
chr5	70951278	70951327	SMN1
chr5	70951525	70951839	SMN1
chr5	70952125	70952175	SMN1
chr5	70952245	70952641	SMN1

chr5	70952704	70953195	SMN1
chr5	70953317	70953941	SMN1
chr5	71604761	71605082	MCCC2
chr5	71605140	71605190	MCCC2
chr5	71605269	71605299	MCCC2
chr5	71605381	71605684	MCCC2
chr5	71606118	71606342	MCCC2
chr5	71606410	71607275	MCCC2
chr5	71607343	71607711	MCCC2
chr5	71607876	71608578	MCCC2
chr5	71608978	71609328	MCCC2
chr5	71609386	71609460	MCCC2
chr5	71609526	71609573	MCCC2
chr5	71609647	71609686	MCCC2
chr5	71609751	71609912	MCCC2
chr5	169665002	169665320	DOCK2
chr5	169821385	169821386	DOCK2
chr5	169821491	169821520	DOCK2
chr5	169823930	169823977	DOCK2
chr5	169824047	169824114	DOCK2
chr5	169824194	169824232	DOCK2
chr5	169825023	169825057	DOCK2
chr5	169825125	169825173	DOCK2
chr5	169834316	169834549	DOCK2
chr5	169997265	169997609	DOCK2
chr5	170004545	170004850	DOCK2
chr5	170004920	170004943	DOCK2
chr6	32038560	32038775	CYP21A2

chr6	32039210	32039225	CYP21A2
chr6	32039316	32039384	CYP21A2
chr6	32039482	32039495	CYP21A2
chr6	32039584	32039632	CYP21A2
chr6	32039730	32039744	CYP21A2
chr6	32040453	32040504	CYP21A2
chr6	32040573	32040635	CYP21A2
chr6	32040697	32040766	CYP21A2
chr6	32040827	32041085	CYP21A2
chr6	32041161	32041430	CYP21A2
chr6	69745327	69745497	LMBRD1
chr6	69749903	69749955	LMBRD1
chr6	69750018	69750075	LMBRD1
chr6	80138798	80138877	BCKDHB
chr6	80138939	80138974	BCKDHB
chr6	80139273	80139365	BCKDHB
chr6	80139428	80139766	BCKDHB
chr6	80139848	80139891	BCKDHB
chr6	80140532	80140567	BCKDHB
chr6	80140631	80140678	BCKDHB
chr6	80140736	80140789	BCKDHB
chr6	80140862	80140916	BCKDHB
chr6	80148999	80149039	BCKDHB
chr6	80149144	80149177	BCKDHB
chr6	80149561	80149766	BCKDHB
chr6	80230091	80230270	BCKDHB
chr6	80239797	80239849	BCKDHB
chr7	44426667	44426684	NUDCD3

chr7	44455836	44455869	NUDCD3
chr7	44455965	44455983	NUDCD3
chr7	66989792	66989852	SBDS
chr7	96140008	96140194	SLC25A13
chr7	96274545	96274567	SLC25A13
chr7	96274663	96274704	SLC25A13
chr7	96275382	96275415	SLC25A13
chr7	117525296	117525990	CFTR
chr7	117526053	117526124	CFTR
chr7	117526185	117529464	CFTR
chr7	117556574	117556759	CFTR
chr7	117610053	117610335	CFTR
chr8	42327870	42328055	IKBKB
chr8	47800152	47800169	PRKDC
chr8	47800239	47800340	PRKDC
chr8	47800403	47800448	PRKDC
chr8	47834749	47834793	PRKDC
chr8	47834871	47834907	PRKDC
chr8	47955242	47955431	PRKDC
chr8	60760308	60760788	CHD7
chr8	60761116	60761354	CHD7
chr9	230486	230513	DOCK8
chr9	230586	230678	DOCK8
chr9	230853	230890	DOCK8
chr9	230952	231171	DOCK8
chr9	231260	231287	DOCK8
chr9	231396	231412	DOCK8
chr9	231507	231563	DOCK8

chr9	232238	232280	DOCK8
chr9	233024	233272	DOCK8
chr9	233340	233395	DOCK8
chr9	233472	233525	DOCK8
chr9	233585	233863	DOCK8
chr9	320302	322414	DOCK8
chr9	380100	380481	DOCK8
chr9	380542	380823	DOCK8
chrX	31184410	31184876	DMD
chrX	31184957	31184973	DMD
chrX	31227724	31227787	DMD
chrX	31227848	31228099	DMD
chrX	31372724	31372726	DMD
chrX	31372824	31372897	DMD
chrX	31372976	31373014	DMD
chrX	31373076	31373387	DMD
chrX	31373480	31373511	DMD
chrX	31373828	31373867	DMD
chrX	31373937	31374338	DMD
chrX	31374610	31374674	DMD
chrX	31511721	31512620	DMD
chrX	31512866	31512883	DMD
chrX	31512968	31513354	DMD
chrX	31530718	31531663	DMD
chrX	31531783	31532171	DMD
chrX	31532248	31535577	DMD
chrX	31559424	31559462	DMD
chrX	31559553	31559580	DMD

chrX	31688746	31688758	DMD
chrX	31689458	31689485	DMD
chrX	31689575	31689631	DMD
chrX	31689735	31689740	DMD
chrX	31690302	31690362	DMD
chrX	31690426	31690481	DMD
chrX	31715323	31715503	DMD
chrX	31749299	31749342	DMD
chrX	31749410	31749562	DMD
chrX	31749625	31749962	DMD
chrX	31750124	31750132	DMD
chrX	31750185	31750422	DMD
chrX	31750619	31751132	DMD
chrX	31897398	31897875	DMD
chrX	31898104	31898117	DMD
chrX	31898203	31898528	DMD
chrX	32244357	32244660	DMD
chrX	32244831	32245188	DMD
chrX	32245282	32246695	DMD
chrX	32400081	32400127	DMD
chrX	32400615	32400907	DMD
chrX	32405902	32405962	DMD
chrX	32406021	32406070	DMD
chrX	32407455	32407522	DMD
chrX	32407629	32407632	DMD
chrX	32486604	32486925	DMD
chrX	32654200	32654234	DMD
chrX	32732231	32732297	DMD

chrX	32732357	32732404	DMD
chrX	32732727	32732768	DMD
chrX	32733134	32733180	DMD
chrX	32733271	32733321	DMD
chrX	32733715	32734156	DMD
chrX	32734213	32734288	DMD
chrX	32734345	32734851	DMD
chrX	32735171	32735198	DMD
chrX	32735269	32735319	DMD
chrX	33118178	33118221	DMD
chrX	33118300	33118352	DMD
chrX	33190536	33190673	DMD
chrX	33304294	33304348	DMD
chrX	33304407	33305253	DMD
chrX	154768767	154768956	DKC1
chrX	154773406	154773447	DKC1
chrX	154773508	154773617	DKC1

Supplementary Table 8. Low coverage regions with 100bp read lengths

Chromosome	Start position	End position	Gene
chr1	33051235	33051790	AK2
chr1	33052124	33052151	AK2
chr1	33052274	33052307	AK2
chr1	33053105	33053410	AK2
chr1	89561160	89561173	LRRC8B
chr1	89561295	89561773	LRRC8B
chr1	89747218	89747733	LRRC8C
chr1	89747867	89747871	LRRC8C

chr1	89748223	89749528	LRRC8C
chr1	89749980	89750861	LRRC8C
chr1	89751097	89751882	LRRC8C
chr1	89752172	89752582	LRRC8C
chr10	74221093	74221185	ADK
chr10	74221668	74221683	ADK
chr11	3898574	3898595	STIM1
chr11	3898706	3898792	STIM1
chr11	3899540	3899594	STIM1
chr11	4001731	4001763	STIM1
chr11	4001853	4001971	STIM1
chr11	4002028	4002120	STIM1
chr11	4003065	4003334	STIM1
chr11	4003394	4003454	STIM1
chr11	4004436	4004718	STIM1
chr11	36551894	36554098	RAG1
chr11	36554200	36557452	RAG1
chr11	108262657	108262915	ATM
chr11	108263293	108263415	ATM
chr11	108263469	108263597	ATM
chr11	108263654	108263756	ATM
chr11	108263861	108263927	ATM
chr11	108264044	108264082	ATM
chr11	108264601	108265093	ATM
chr11	108265531	108265930	ATM
chr11	108358354	108358380	ATM
chr11	108358515	108358551	ATM
chr11	108359092	108359147	ATM

chr11	108359226	108359259	ATM
chr11	108360366	108360387	ATM
chr11	108360732	108360733	ATM
chr11	108360849	108360955	ATM
chr11	108361019	108361374	ATM
chr11	108361909	108362253	ATM
chr11	108362503	108362542	ATM
chr13	100319338	100319362	PCCA
chr15	76257628	76257681	ETFA
chr15	82242324	82243758	EFL1
chr15	82250121	82250561	EFL1
chr16	30188630	30189075	CORO1A
chr17	42219350	42219735	STAT5B
chr17	42264717	42265112	STAT5B
chr2	210507693	210507698	CPS1
chr2	210666224	210666326	CPS1
chr2	210666386	210666475	CPS1
chr2	216459640	216459661	SMARCAL1
chr21	43053191	43055720	CBS
chr21	43055816	43056597	CBS
chr21	43056696	43057237	CBS
chr21	43057517	43059956	CBS
chr21	43060053	43061733	CBS
chr21	43061830	43063613	CBS
chr21	43063711	43065194	CBS
chr21	43065289	43066071	CBS
chr21	43066303	43066769	CBS
chr21	43067219	43067294	CBS

chr21	43067393	43067728	CBS
chr21	43067976	43068059	CBS
chr21	43068149	43068238	CBS
chr21	43068332	43068424	CBS
chr21	43068532	43068563	CBS
chr21	43068812	43070057	CBS
chr21	43070151	43070898	CBS
chr21	43070969	43075908	CBS
chr21	43075999	43076781	CBS
chr5	70049612	70052156	SMN2
chr5	70052237	70052856	SMN2
chr5	70052927	70060210	SMN2
chr5	70060310	70068335	SMN2
chr5	70068411	70068730	SMN2
chr5	70068820	70070126	SMN2
chr5	70070195	70071135	SMN2
chr5	70071233	70072838	SMN2
chr5	70072936	70074566	SMN2
chr5	70075118	70075447	SMN2
chr5	70076188	70076385	SMN2
chr5	70076862	70077204	SMN2
chr5	70077303	70077750	SMN2
chr5	70077928	70078521	SMN2
chr5	70925030	70927577	SMN1
chr5	70927655	70928273	SMN1
chr5	70928353	70935634	SMN1
chr5	70935729	70943759	SMN1
chr5	70943835	70944153	SMN1

chr5	70944253	70945554	SMN1
chr5	70945617	70946562	SMN1
chr5	70946654	70947101	SMN1
chr5	70947201	70948266	SMN1
chr5	70948365	70949990	SMN1
chr5	70950541	70950873	SMN1
chr5	70951553	70951808	SMN1
chr5	70952284	70952620	SMN1
chr5	70952727	70953164	SMN1
chr5	70953349	70953941	SMN1
chr5	71604774	71605054	MCCC2
chr5	71606480	71606943	MCCC2
chr5	71607012	71607065	MCCC2
chr5	71607203	71607232	MCCC2
chr5	71607357	71607433	MCCC2
chr5	71607496	71607571	MCCC2
chr5	71607990	71608552	MCCC2
chr5	169665021	169665300	DOCK2
chr5	169997471	169997484	DOCK2
chr5	170004583	170004608	DOCK2
chr5	170004717	170004810	DOCK2
chr6	32040842	32040909	CYP21A2
chr6	32041006	32041067	CYP21A2
chr6	32041177	32041266	CYP21A2
chr6	32041374	32041407	CYP21A2
chr6	80139299	80139336	BCKDHB
chr7	117525323	117525835	CFTR
chr7	117525893	117525969	CFTR

chr7	117526313	117527972	CFTR
chr7	117528086	117529193	CFTR
chr7	117529249	117529351	CFTR
chr8	60760339	60760736	CHD7
chr9	320318	322382	DOCK8
chr9	380120	380468	DOCK8
chr9	380557	380659	DOCK8
chr9	380766	380791	DOCK8
chrX	31184445	31184720	DMD
chrX	31228017	31228067	DMD
chrX	31511828	31511947	DMD
chrX	31512285	31512593	DMD
chrX	31513009	31513017	DMD
chrX	31513096	31513232	DMD
chrX	31513301	31513326	DMD
chrX	31530748	31530750	DMD
chrX	31530867	31531551	DMD
chrX	31531823	31532110	DMD
chrX	31532311	31535547	DMD
chrX	31749645	31749725	DMD
chrX	31750231	31750265	DMD
chrX	31750752	31750806	DMD
chrX	31897426	31897773	DMD
chrX	31898232	31898266	DMD
chrX	32244412	32244569	DMD
chrX	32244970	32245030	DMD
chrX	32245434	32245899	DMD
chrX	32245993	32246079	DMD

chrX	32246172	32246282	DMD
chrX	32246353	32246693	DMD
chrX	32486651	32486669	DMD
chrX	32486803	32486826	DMD
chrX	32733774	32733805	DMD
chrX	32733900	32733996	DMD
chrX	32734080	32734127	DMD
chrX	33304619	33304927	DMD
chrX	33305004	33305072	DMD

Supplementary Table 9. Low coverage regions with 150bp read lengths

Chromosome	Start position	End position	Gene
chr1	33051288	33051774	AK2
chr1	89561538	89561589	LRRC8B
chr1	89748321	89748327	LRRC8C
chr1	89748379	89748654	LRRC8C
chr1	89748994	89749358	LRRC8C
chr1	89750291	89750414	LRRC8C
chr1	89750530	89750655	LRRC8C
chr1	89751244	89751248	LRRC8C
chr1	89751419	89751490	LRRC8C
chr1	89752300	89752308	LRRC8C
chr11	36551972	36552587	RAG1
chr11	36552746	36552840	RAG1
chr11	36552911	36553101	RAG1
chr11	36553160	36553298	RAG1
chr11	36553386	36554081	RAG1

chr11	36554244	36557389	RAG1
chr11	108264767	108264917	ATM
chr15	82242400	82243674	EFL1
chr15	82250202	82250516	EFL1
chr16	30188728	30189075	CORO1A
chr17	42219412	42219691	STAT5B
chr17	42264911	42264924	STAT5B
chr21	43053191	43055676	CBS
chr21	43055856	43056562	CBS
chr21	43056737	43057192	CBS
chr21	43057577	43059913	CBS
chr21	43060101	43061691	CBS
chr21	43061880	43063564	CBS
chr21	43063746	43065148	CBS
chr21	43065328	43066021	CBS
chr21	43066359	43066711	CBS
chr21	43067433	43067447	CBS
chr21	43068858	43070013	CBS
chr21	43070192	43070869	CBS
chr21	43070991	43075864	CBS
chr21	43076037	43076740	CBS
chr5	70049612	70052130	SMN2
chr5	70052263	70052828	SMN2
chr5	70052955	70060170	SMN2
chr5	70060351	70068306	SMN2
chr5	70068871	70070101	SMN2
chr5	70070219	70071084	SMN2
chr5	70071281	70071380	SMN2

chr5	70071518	70071632	SMN2
chr5	70071820	70072794	SMN2
chr5	70072984	70074483	SMN2
chr5	70077095	70077166	SMN2
chr5	70077345	70077707	SMN2
chr5	70077980	70078521	SMN2
chr5	70925030	70927548	SMN1
chr5	70927685	70928247	SMN1
chr5	70928380	70935584	SMN1
chr5	70935779	70943732	SMN1
chr5	70944292	70945533	SMN1
chr5	70945642	70946517	SMN1
chr5	70946696	70947062	SMN1
chr5	70947237	70948221	SMN1
chr5	70948407	70949916	SMN1
chr5	70952508	70952589	SMN1
chr5	70952758	70953111	SMN1
chr5	70953392	70953941	SMN1
chr5	71606720	71606807	MCCC2
chr7	117525373	117525771	CFTR
chr7	117526396	117527914	CFTR
chr7	117528753	117528960	CFTR
chr7	117529021	117529053	CFTR
chr9	320544	320643	DOCK8
chr9	320835	322329	DOCK8
chr9	380340	380424	DOCK8
chrX	31532381	31533101	DMD
chrX	31533170	31533960	DMD

chrX	31534050	31534726	DMD
chrX	31534862	31534943	DMD
chrX	31535064	31535496	DMD
chrX	32246452	32246465	DMD

Supplementary Table 10. Low coverage regions with 250bp read lengths

Chromosome	Start position	End position	Gene
chr11	36552099	36552121	RAG1
chr11	36552380	36552483	RAG1
chr11	36553558	36553604	RAG1
chr11	36553852	36553908	RAG1
chr11	36554638	36554863	RAG1
chr11	36555074	36556734	RAG1
chr15	82242502	82243616	EFL1
chr16	30188845	30189075	CORO1A
chr21	43053191	43055584	CBS
chr21	43055963	43056462	CBS
chr21	43057672	43059824	CBS
chr21	43060178	43061608	CBS
chr21	43061958	43063469	CBS
chr21	43063843	43065040	CBS
chr21	43065420	43065920	CBS
chr21	43069035	43069932	CBS
chr21	43070289	43070780	CBS
chr21	43071089	43075755	CBS
chr21	43076150	43076614	CBS
chr5	70049612	70052041	SMN2
chr5	70053041	70060083	SMN2
chr5	70060453	70068209	SMN2
chr5	70068965	70070008	SMN2
chr5	70070307	70070991	SMN2

chr5	70071901	70072684	SMN2
chr5	70073092	70074393	SMN2
chr5	70078088	70078521	SMN2
chr5	70925030	70927450	SMN1
chr5	70928487	70935489	SMN1
chr5	70935862	70943624	SMN1
chr5	70944391	70945440	SMN1
chr5	70945737	70946415	SMN1
chr5	70947330	70948132	SMN1
chr5	70948492	70949805	SMN1
chr5	70953493	70953941	SMN1
chr7	117526584	117527750	CFTR
chrX	31532471	31532957	DMD
chrX	31533301	31533780	DMD

Supplementary Table 11. Variant calling results for simulated homozygous pathogenic variants on CYP21A2 with MQ≥20 filter.

Variant	Position	REF	ALT	Called Position	Called REF	Called ALT	GT
**1**	chr6:32039081	C	G	32039081	C	G	1/1
**2**	chr6:32038514	C	T	32038514	C	T	1/1
**3**	chr6:32039162	A	C	32039162	A	C	1/1
**4**	chr6:32039426	T	A	32039426	T	A	1/1
**5**	chr6:32040110	G	C	32040110	G	C	1/1
**6**	chr6:32040110	G	T	32040110	G	T	1/1
**7**	chr6:32040140	G	A	32040140	G	A	1/1
**8**	chr6:32040421	C	T	32040421	C	T	1/1
**9**	chr6:32040535	C	T	No Call	No Call	No Call	No Call
**10**	chr6:32040766	G	A	32040766	G	A	1/1
**11**	chr6:32041006	C	T	32041006	C	T	1/1

Supplementary Table 12. Variant calling results for simulated heterozygous pathogenic variants on CYP21A2 with MQ ≥ 20 filter.

Variant	Position	REF	ALT	Called Position	Called REF	Called ALT	GT
1	chr6:32039081	C	G	32039081	C	G	0/1
2	chr6:32038514	C	T	32038514	C	T	0/1
3	chr6:32039162	A	C	32039162	A	C	0/1
4	chr6:32039426	T	A	32039426	T	A	0/1
5	chr6:32040110	G	C	32040110	G	C	0/1
6	chr6:32040110	G	T	32040110	G	T	0/1
7	chr6:32040140	G	A	32040140	G	A	0/1
8	chr6:32040421	C	T	32040421	C	T	0/1
9	chr6:32040535	C	T	32040535	No call	No call	No call
10	chr6:32040766	G	A	32040766	G	A	0/1
11	chr6:32041006	C	T	32041006	C	T	0/1

Supplementary Table 13. Variant calling results for simulated homozygous pathogenic variants on CYP21A2 with MQ ≥ 10 filter.

Variant	Position	REF	ALT	Called Position	Called REF	Called ALT	GT
1	chr6:32039081	C	G	32039081	C	G	1/1
2	chr6:32038514	C	T	32038514	C	T	1/1
3	chr6:32039162	A	C	32039162	A	C	1/1
4	chr6:32039426	T	A	32039426	T	A	1/1
5	chr6:32040110	G	C	32040110	G	C	1/1
6	chr6:32040110	G	T	32040110	G	T	1/1
7	chr6:32040140	G	A	32040140	G	A	1/1
8	chr6:32040421	C	T	32040421	C	T	1/1
9	chr6:32040535	C	T	No Call	No Call	No Call	No Call

10	chr6:32040766	G	A	32040766	G	A	1/1
11	chr6:32041006	C	T	32041006	C	T	1/1

Supplementary Table 14. Variant calling results for all simulated homozygous pathogenic variants on *CYP21A2* with *CYP21A1P* masked and run with ploidy = 4.

Variant	Position	REF	ALT	Called Position	Called REF	Called ALT	GT
1	chr6:32039081	C	G	32039081	C	G	0/0/1/1
2	chr6:32038514	C	T	32038514	C	T	0/0/1/1
3	chr6:32039162	A	C	32039162	A	C	0/0/1/1
4	chr6:32039426	T	A	32039426	T	A	0/0/1/1
5	chr6:32040110	G	C	32040110	G	C,T	1/1/2/2
6	chr6:32040110	G	T	32040110	G	T	0/0/1/1
7	chr6:32040140	G	A	32040140	G	A	0/0/1/1
8	chr6:32040421	C	T	32040421	C	T	0/0/1/1
9	chr6:32040535	C	T	32040535	C	T	1/1/1/1
10	chr6:32040766	G	A	2040766	G	A	0/1/1/1
11	chr6:32041006	C	T	32041006	C	T	1/1/1/1

Supplementary Table 15. Variants called on *CYP21A2* when variant called with ploidy = 4 on the *CYP21A1P* masked reference genome when homozygous variant chr6:32040535 is simulated.

Chromosome	Position	Called REF	Called ALT	GT
**chr6**	32038419	C	T	0/0/0/1
**chr6**	32038514	C	T	0/0/1/1
**chr6**	32038844	A	C	0/0/1/1
**chr6**	32038855	T	TTTG	0/0/1/1
**chr6**	32038857	A	T	0/0/1/1
**chr6**	32038867	T	G	0/0/1/1

**chr6**	32038878	C	T	0/0/1/1
**chr6**	32038895	A	G	0/0/1/1
**chr6**	32038903	A	G	0/0/1/1
**chr6**	32038911	A	G	0/0/1/1
**chr6**	32038927	A	G	0/0/1/1
**chr6**	32038938	T	G	0/0/1/1
**chr6**	32038948	T	TC	0/0/1/1
**chr6**	32038949	T	G	0/0/1/1
**chr6**	32038955	A	T	0/0/1/1
**chr6**	32038962	A	ATCC	0/0/1/1
**chr6**	32038971	C	A	0/0/1/1
**chr6**	32038983	TG	T	0/0/1/1
**chr6**	32038997	TGG	T	0/0/1/1
**chr6**	32039001	G	C	0/0/1/1
**chr6**	32039002	G	A	0/0/1/1
**chr6**	32039007	A	AAG	0/0/1/1
**chr6**	32039015	G	T	0/0/1/1
**chr6**	32039020	G	A	0/0/1/1
**chr6**	32039027	C	A	0/0/1/1
**chr6**	32039046	A	G	0/0/1/1
**chr6**	32039050	G	T	0/0/1/1
**chr6**	32039055	C	G	0/0/1/1
**chr6**	32039056	A	G	0/0/1/1
**chr6**	32039081	C	G	0/0/1/1
**chr6**	32039109	G	A	0/0/1/1
**chr6**	32039128	C	G	0/0/1/1
**chr6**	32039132	GGAGACTAC	G	0/0/1/1
**chr6**	32039143	C	T	0/0/1/1

**chr6**	32039426	T	A	0/0/1/1
**chr6**	32039531	C	A	0/0/1/1
**chr6**	32039538	T	C	0/0/1/1
**chr6**	32039548	C	G	0/0/1/1
**chr6**	32039802	T	C	0/0/1/1
**chr6**	32039807	T	A	0/0/1/1
**chr6**	32039810	T	A	0/0/1/1
**chr6**	32039816	T	A	0/0/1/1
**chr6**	32039847	A	G	0/0/1/1
**chr6**	32039848	C	T	0/0/1/1
**chr6**	32040013	C	G	0/0/1/1
**chr6**	32040110	G	T	0/0/1/1
**chr6**	32040182	G	GT	0/0/1/1
**chr6**	32040216	G	C	0/0/1/1
**chr6**	32040421	C	T	0/0/1/1
**chr6**	32040535	C	T	1/1/1/1
**chr6**	32040797	G	A	0/0/0/1

Supplementary Table 16. Variant calling results for simulated homozygous pathogenic variants on *CYP21A2* with MQ ≥ 20 filter and large inner distance between reads.

Variant	Position	REF	ALT	Called Position	Called REF	Called ALT	GT
1	chr6:32039081	C	G	32039081	C	G	1/1
2	chr6:32038514	C	T	32038514	C	T	1/1
3	chr6:32039162	A	C	32039162	A	C	1/1
4	chr6:32039426	T	A	32039426	T	A	1/1
5	chr6:32040110	G	C	32040110	G	C	1/1
6	chr6:32040110	G	T	32040110	G	T	1/1

7	chr6:32040140	G	A	32040140	G	A	1/1
8	chr6:32040421	C	T	32040421	C	T	1/1
9	chr6:32040535	C	T	32040535	C	T	1/1
10	chr6:32040766	G	A	2040766	G	A	1/1
11	chr6:32041006	C	T	32041006	C	T	1/1

Supplementary Table 17. Variant calling results for simulated homozygous variants on *SMN1, CORO1A* and *CBS* with the standard GATK variant calling pipeline, increased inner distance between reads and increased ploidy with masking of homologous regions.

Gene	Chr	Position	REF Allele	ALT Allele	Pathogenicity	Standard Variant Calling Result	Increased Inner Distance Result	Increased Ploidy Result
SMN1	5	70925108	C	G	Pathogenic	No call	No call	No call*
SMN1	5	70942416	C	G	Pathogenic	No call	No call	0/1/1/1*
SMN1	5	70944713	T	A	Pathogenic	No call	No call	No call
SMN1	5	70942553	C	T	Pathogenic	No call	No call	0/0/1/1
SMN1	5	70946127	G	T	Pathogenic	No call	No call	No call*
CORO1A	16	30188946	G	A	Likely benign	No call	No call	No call
CBS	21	43058970	T	G	Pathogenic	No call	No call	0/0/1/1*
CBS	21	43059299	T	C	Pathogenic	No call	No call	No call
CBS	21	43060475	C	T	Pathogenic	No call	No call	No call
CBS	21	43065684	C	T	Pathogenic	No call	No call	No call
CBS	21	43072048	G	A	Pathogenic	No call	No call	No call

*Results for the given position, however other variants in different positions were also called.

Supplementary Table 18. NBS gene list with identification (ID) codes, chromosome positioning on GRCh38 according to the ENSEMBL 94 annotation set and associated NBS disease.

Gene Name	HGNC ID	Chromosome	Start	End	NBS Disease
ABCD1	HGNC:61	X	153724868	153744762	X-linked Adrenoleukodystrophy
ACAD8	HGNC:87	11	134253495	134265855	Isovaleric Acidemia
ACADM	HGNC:89	1	75724347	75787575	Medium-chain Acyl-CoA Dehydrogenase Deficiency (MCAD)
ACADS	HGNC:90	12	120725735	120740008	Medium-chain Acyl-CoA Dehydrogenase Deficiency (MCAD)
ACADSB	HGNC:91	10	123008979	123058311	3-Hydroxy-3-Methylglutaric Aciduria
ACADVL	HGNC:92	17	7217125	7225273	Very Long-chain L-3-Hydroxyacyl-CoA Dehydrogenase Deficiency (VLCAD)
ACAT1	HGNC:93	11	108121516	108147776	B-Ketothiolase Deficiency (BKT)
ADA	HGNC:186	20	44619522	44652233	SCID, T cell deficiency and IEM
ADK	HGNC:257	10	74151185	74709303	Hypermethioninemia
AHCY	HGNC:343	20	34280268	34311802	Hypermethioninemia
AK2	HGNC:362	1	33007940	33080996	SCID and T cell defects
ALDH7A1	HGNC:877	5	126531200	126595418	B6 Responsive Seizures
ARG1	HGNC:663	6	131573144	131584332	Argininemia
ARPC1B	HGNC:704	7	99374249	99394816	SCID and T cell defects
ARSA	HGNC:713	22	50622754	50628173	Metaomatic Leukodystrophy (MLD)
ASL	HGNC:746	7	66075798	66093558	Argininosuccinic aciduria
ASS1	HGNC:758	9	130444929	130501274	Citrullinemia Type I
ATM	HGNC:795	11	108222484	108369102	T cell defect, Ataxia Telangiectasia, low TRECs[1]
BCKDHA	HGNC:986	19	41397460	41425005	Maple Syrup Urine Disease (MSUD)

BCKDHB	HGNC:987	6	80106647	80346270	Maple Syrup Urine Disease (MSUD)
BCKDK	HGNC:16902	16	31106107	31112791	Branched-Chain Keto Acid Dehydrogenase Kinase Deficiency
BCL11B	HGNC:13222	14	99169287	99271524	Severe Combined Immunodeficiencies (SCID)
BLNK	HGNC:14211	10	96191702	96271587	Antibody deficiency, low KRECs[2]
BTD	HGNC:1122	3	15601341	15647640	Biotinidase Deficiency (BTD)
BTK	HGNC:1133	X	101349447	101390796	Antibody deficiency, low KRECs[2]
CARMIL2	HGNC:27089	16	67644919	67657569	Combined Immunodeficiency
CBS	HGNC:1550	21	43053191	43076943	Homocystinuria (HCY)
CD247	HGNC:1677	1	167430640	167518610	Combined Immunodeficiency
CD3D	HGNC:1673	11	118338954	118342744	SCID and T cell defects
CD3E	HGNC:1674	11	118304545	118316175	SCID and T cell defects
CD3G	HGNC:1675	11	118344344	118355161	SCID and T cell defects
CD40LG	HGNC:11935	X	136648193	136660390	Antibody deficiency, low KRECs[2]
CD79A	HGNC:1698	19	41877120	41881372	SCID and T cell defects
CD79B	HGNC:1699	17	63928740	63932354	Antibody deficiency, low KRECs[2]
CD8A	HGNC:1706	2	86784610	86808396	SCID and T cell defects
CFTR	HGNC:1884	7	117465784	117715971	Cystic Fibrosis (CF)
CHD7	HGNC:20626	8	60678778	60868028	CHARGE syndrome, thymic aplasia, low TRECs[1]
CORO1A	HGNC:2252	16	30182827	30189076	SCID and T cell defects
CPS1	HGNC:2323	2	210477682	210679107	Carbamoyl-phosphate Synthase Deficiency
CPT1A	HGNC:2328	11	68754620	68844410	Carnitine 1 Uptake Defect (CPT1)
CPT2	HGNC:2330	1	53196429	53214197	Carnitine 2 Uptake Defect (CPT1)

CYP11A1	HGNC:2590	15	74337759	74367740	Congenital Adrenal Hyperplasia (CAH)
CYP17A1	HGNC:2593	10	102830531	102837533	Congenital Adrenal Hyperplasia (CAH)
CYP21A2	HGNC:2600	6	32038265	32041670	Congenital Adrenal Hyperplasia (CAH)
DBT	HGNC:2698	1	100186919	100249834	Maple Syrup Urine Disease (MSUD)
DCLRE1C	HGNC:17642	10	14897359	14954432	SCID and T cell defects
DKC1	HGNC:2890	X	154762742	154777689	Combined Immunodeficiency, Dyskeratosis Congenita
DLD	HGNC:2898	7	107890970	107931730	Maple Syrup Urine Disease (MSUD)
DMD	HGNC:2928	X	31097677	33339441	Duchenne Muscular Dystrophy
DNMT3B	HGNC:2979	20	32762385	32809356	SCID and T cell defects
DOCK2	HGNC:2988	5	169637247	170083382	SCID and T cell defects
DOCK8	HGNC:19191	9	214854	465259	Combined Immunodeficiency
EFL1	HGNC:25789	15	82130230	82262763	SCID and T cell defects
ETFA	HGNC:3481	15	76215355	76311472	Glutaric Acidemia Type II (GA2)
ETFB	HGNC:3482	19	51345169	51366418	Glutaric Acidemia Type II (GA2)
ETFDH	HGNC:3483	4	158672125	158709623	Glutaric Acidemia Type II (GA2)
EXTL3	HGNC:3518	8	28600469	28755599	SCID and T cell defects
FAH	HGNC:3579	15	80152490	80186946	Tyrosinemia Type I (TYR1)
FOXN1	HGNC:12765	17	28506243	28538896	SCID and T cell defects
GAA	HGNC:4065	17	80101556	80119879	Glycogen Storage Disease Type II (GSD II)
GALE	HGNC:4116	1	23795599	23800804	Galactoepimerase Deficiency
GALK1	HGNC:4118	17	75751594	75765711	Galactokinase Deficiency
GALT	HGNC:4135	9	34638133	34651035	Classic Galactosemia

GAMT	HGNC:4136	19	1397026	1401570	Cerebral Creatine Deficiency Syndrome 2
GATA2	HGNC:4171	3	128479427	128493185	Combined Immunodeficiency
GATM	HGNC:4175	15	45361124	45402327	Cerebral Creatine Deficiency Syndrome 3
GCDH	HGNC:4189	19	12891026	12914207	Glutaric Acidemia Type I (GA1)
HADHA	HGNC:4801	2	26190635	26244726	Trifunctional Protein Deficiency (TFP) / Long-chain L-3-Hydroxyacyl-CoA Dehydrogenase Deficiency (LCHAD)
HADHB	HGNC:4803	2	26243170	26290468	Trifunctional Protein Deficiency (TFP)
HBB	HGNC:4827	11	5225464	5229395	Sickle Cell Anemia / Beta-Thalassemia
HLCS	HGNC:4976	21	36750888	36990236	Holocarboxylase Synthase Deficiency
HMGCL	HGNC:5005	1	23801885	23838620	3-Hydroxy-3-Methylglutaric Aciduria
HMGCS2	HGNC:5008	1	119747996	119768905	3-Hydroxy-3-Methyglutaric Aciduria
HPD	HGNC:5147	12	121839527	121863596	Tyrosinemia Type III (TYR III)
HPGD	HGNC:5154	4	174490177	174523154	Not in NBS gene panel. Weak indication of NBS interest.
IDUA	HGNC:5391	4	986997	1004506	Mucopolysaccharidosis Type 1 (MPS I)
IGHM	HGNC:5541	14	105851705	105856218	Antibody deficiency, low KRECs
IGLL1	HGNC:5870	22	23573125	23580308	Antibody deficiency, low KRECs[2]
IKBKB	HGNC:5960	8	42271302	42332653	Combined Immunodeficiency
IKZF1	HGNC:13176	7	50304068	50405101	SCID and T cell defects
IL2RG	HGNC:6010	X	71107404	71112108	SCID and T cell defects
IL7R	HGNC:6024	5	35852695	35879603	SCID and T cell defects

IVD	HGNC:6186	15	40405795	40435947	Isovaleric Acidemia (IVA)
JAK3	HGNC:6193	19	17824780	17848071	Severe Combined Immunodeficiencies (SCID)
LAT	HGNC:18874	16	28984826	28990783	SCID and T cell defects
LCK	HGNC:6524	1	32251239	32286165	Combined Immunodeficiency
LIG4	HGNC:6601	13	108207439	108218368	SCID and T cell defects
LMBRD1	HGNC:23038	6	69675793	69867236	Methylmalonic Acidemia (MMA)
LRRC8A	HGNC:19027	9	128882112	128918039	Antibody deficiency, low KRECs[2]
LRRC8B	HGNC:30692	1	89524836	89597864	LRRC8A paralog. Not in NBS gene panel. Weak indication of NBS interest.
LRRC8C	HGNC:25075	1	89633072	89769903	LRRC8A paralog. Not in NBS gene panel. Weak indication of NBS interest.
LRRC8D	HGNC:16992	1	89821014	89936611	LRRC8A paralog. Not in NBS gene panel. Weak indication of NBS interest.
LRRC8E	HGNC:26272	19	7888505	7902021	LRRC8A paralog. Not in NBS gene panel. Weak indication of NBS interest.
MAT1A	HGNC:6903	10	80271820	80289684	Hypermethioninemia
MC2R	HGNC:6930	18	13882044	13915707	Congenital Adrenal Hyperplasia (CAH)
MCCC1	HGNC:6936	3	183015218	183116075	3-Methylcrotonyl-CoA Carboxylase Deficiency
MCCC2	HGNC:6937	5	71587288	71658704	3-Methylcrotonyl-CoA Carboxylase Deficiency
MMAA	HGNC:18871	4	145599042	145660035	Methylmalonic Acidemia (MMA)
MMAB	HGNC:19331	12	109553737	109573874	Methylmalonic Acidemia (MMA)
MMACHC	HGNC:24525	1	45500053	45513382	Methylmalonic Acidemia (MMA)/Homocystinuria (HCY)
MMADHC	HGNC:25221	2	149569634	149587816	Methylmalonic Acidemia (MMA)/Homocystinuria (HCY)

MTHFR	HGNC:7436	1	11785723	11806920	Homocystinuria (HCY)
MTR	HGNC:7468	1	236795281	236903981	Homocystinuria (HCY)
MTRR	HGNC:7473	5	7851186	7906025	Homocystinuria (HCY)
MUT	HGNC:7526	6	49430360	49463191	Methylmalonic Acidemia (MMA)
NADK2	HGNC:26404	5	36192592	36242279	2,4 Dienoyl-CoA Reductase Deficiency
NAGS	HGNC:17996	17	44004546	44009063	Ornithine Transcarbamylase Deficiency
NBN	HGNC:7652	8	89933336	90003228	T cell defect, Nijmegen breakage syndrome, low TRECs[1]
NHEJ1	HGNC:25737	2	219075317	219160865	SCID and T cell defects
NPC1	HGNC:7897	18	23506184	23586898	Niemann–Pick Disease Type C1
NPC2	HGNC:14537	14	74476192	74494177	Niemann–Pick Disease Type C2
NUDCD3	HGNC:22208	7	44379121	44490880	Not in NBS gene panel. Weak indication of NBS interest.
OTC	HGNC:8512	X	38352545	38421450	Ornithine Transcarbamylase Deficiency
PCBD1	HGNC:8646	10	70882280	70888784	Hyperphenylalaninemia
PCCA	HGNC:8653	13	100089015	100530437	Propionic Acidemia (PKU)
PCCB	HGNC:8654	3	136250306	136337896	Propionic Acidemia (PKU)
PGM3	HGNC:8907	6	83161150	83193936	SCID, T cell deficiency and IEM
PIK3CD	HGNC:8977	1	9651732	9729114	Combined Immunodeficiency
PIK3R1	HGNC:8979	5	68215720	68301821	Antibody deficiency, low KRECs[2]
PAH	HGNC:8582	12	102836885	102958410	Phenylketonuria (PKU)
PAX1	HGNC:8615	20	21705659	21718486	Otofaciocervical syndrome, thymic aplasia, low TRECs[1]
PNP	HGNC:7892	14	20468954	20477094	SCID, T cell deficiency and IEM

POR	HGNC:9208	7	75899200	75986855	Congenital Adrenal Hyperplasia (CAH)
PRKDC	HGNC:9413	8	47773108	47960183	Combined Immunodeficiency
PSAP	HGNC:9498	10	71816298	71851375	Metaomatic Leukodystrophy (MLD)
PSAT1	HGNC:19129	9	78297143	78330093	Neu-Laxova syndrome 2, differention to PSPH deficiency
PSPH	HGNC:9577	7	56011051	56051604	Phosphoserine Phosphatase Deficiency
PTPRC	HGNC:9666	1	198638457	198757476	Combined Immunodeficiency)
PTS	HGNC:9689	11	112226365	112269955	Hyperphenylalaninemia
QDPR	HGNC:9752	4	17460261	17512234	Hyperphenylalaninemia
RAC2	HGNC:9802	22	37225261	37244448	Combined Immunodeficiency
RAG1	HGNC:9831	11	36510709	36593156	SCID and T cell defects
RAG2	HGNC:9832	11	36575574	36598279	SCID and T cell defects
RECQL4	HGNC:9949	8	144511288	144517845	SCID and T cell defects
RMRP	HGNC:10031	9	35657754	35658017	SCID and T cell defects
SBDS	HGNC:19440	7	66987677	66995601	SCID and T cell defects)
SLC16A1	HGNC:10922	1	112911847	112957013	Monocarboxylate Transporter 1 Deficiency
SLC22A5	HGNC:10969	5	132369752	132395614	Carnitine Uptake Defect (CUD)
SLC25A13	HGNC:10983	7	96120220	96322147	Citrullinemia Type II
SLC25A15	HGNC:10985	13	40789412	40810111	Hyperornithinemia-Hyperammonemia-Homocitrullinuria Syndrome
SLC25A20	HGNC:1421	3	48856931	48898993	Carnitine Acylcarnitine Translocase Deficiency (CACT)
SLC46A1	HGNC:30521	17	28394756	28407197	SCID and T cell defects
SLC52A2	HGNC:30224	8	144354135	144361286	Brown-Vialetto-Van Laere syndrome 2,Vitamin B2 responsive

SLC52A3	HGNC:16187	20	760080	776015	Brown-Vialetto-Van Laere syndrome 1, Vitamin B2 responsive
SLC6A8	HGNC:11055	X	153688099	153696593	Cerebral Creatine Deficiency Syndrome 1
SMARCAL1	HGNC:11102	2	216412414	216483053	Combined Immunodeficiency
SMN1	HGNC:11117	5	70925030	70953942	Spinal Muscular Atrophy (SMA)
SMN2	HGNC:11118	5	70049612	70078522	Spinal Muscular Atrophy (SMA)
STAT5B	HGNC:11367	17	42199168	42276707	SCID and T cell defects
STIM1	HGNC:11386	11	3854527	4093210	SCID and T cell defects
TAT	HGNC:11573	16	71565660	71577130	Tyrosinemia Type II (TYR II)
TBX1	HGNC:11592	22	19756703	19783593	DiGeorge syndrome, thymic aplasia, low TRECs[1]
TCF3	HGNC:11633	19	1609290	1652605	Antibody deficiency, low KRECs[2], Only 1 variant: NM_001136139.2(TCF3): c.1663G>A (p.Glu555Lys)
TTC7A	HGNC:19750	2	46916157	47076137	SCID and T cell defects
UNC119	HGNC:12565	17	28546707	28552668	Antibody deficiency, low KRECs[2]
WAS	HGNC:12731	X	48676596	48691427	Combined Immunodeficiency
ZAP70	HGNC:12858	2	97713560	97739862	SCID and T cell defects
ZBTB24	HGNC:21143	6	109462594	109483237	SCID and T cell defects

[1] T cell receptor excision circles (TRECs) can be used to identify impaired T cell development in primary immunodeficiencies.
[2] kappa-deleting recombination excision circles (KRECs) can be used to detect impaired B cell development in primary immunodeficiencies.

Supplementary Table 19. Sample information for simulated genomes.

Sample ID	Population Code	Location	Superpopulation Code	Superpopulation
NA20882	GIH	Gujarati Indian in Houston, TX	SAS	South Asian
NA20854	GIH	Gujarati Indian in Houston, TX	SAS	South Asian
NA20856	GIH	Gujarati Indian in Houston, TX	SAS	South Asian
NA20862	GIH	Gujarati Indian in Houston, TX	SAS	South Asian
NA20868	GIH	Gujarati Indian in Houston, TX	SAS	South Asian
NA20874	GIH	Gujarati Indian in Houston, TX	SAS	South Asian
NA20876	GIH	Gujarati Indian in Houston, TX	SAS	South Asian
NA20881	GIH	Gujarati Indian in Houston, TX	SAS	South Asian
NA20899	GIH	Gujarati Indian in Houston, TX	SAS	South Asian
NA20902	GIH	Gujarati Indian in Houston, TX	SAS	South Asian
HG00174	FIN	Finland	EUR	European
HG00176	FIN	Finland	EUR	European
HG00272	FIN	Finland	EUR	European
HG00276	FIN	Finland	EUR	European
HG00288	FIN	Finland	EUR	European
HG00309	FIN	Finland	EUR	European
HG00331	FIN	Finland	EUR	European
HG00334	FIN	Finland	EUR	European
HG00377	FIN	Finland	EUR	European
HG00378	FIN	Finland	EUR	European
HG00404	CHS	Han Chinese in Beijing, China	EAS	East Asian

HG00419	CHS	Han Chinese in Beijing, China	EAS	East Asian
HG00437	CHS	Han Chinese in Beijing, China	EAS	East Asian
HG00531	CHS	Han Chinese in Beijing, China	EAS	East Asian
HG00566	CHS	Han Chinese in Beijing, China	EAS	East Asian
HG00608	CHS	Han Chinese in Beijing, China	EAS	East Asian
HG00632	CHS	Han Chinese in Beijing, China	EAS	East Asian
HG00654	CHS	Han Chinese in Beijing, China	EAS	East Asian
HG00675	CHS	Han Chinese in Beijing, China	EAS	East Asian
HG00428	CHS	Han Chinese in Beijing, China	EAS	East Asian
HG01119	CLM	Colombians from Medellin, Colombia	AMR	Admixed American
HG01251	CLM	Colombians from Medellin, Colombia	AMR	Admixed American
HG01345	CLM	Colombians from Medellin, Colombia	AMR	Admixed American
HG01366	CLM	Colombians from Medellin, Colombia	AMR	Admixed American
HG01375	CLM	Colombians from Medellin, Colombia	AMR	Admixed American
HG01390	CLM	Colombians from Medellin, Colombia	AMR	Admixed American
HG01444	CLM	Colombians from Medellin, Colombia	AMR	Admixed American
HG01456	CLM	Colombians from Medellin, Colombia	AMR	Admixed American
HG01468	CLM	Colombians from Medellin, Colombia	AMR	Admixed American
HG01134	CLM	Colombians from Medellin, Colombia	AMR	Admixed American
HG02646	GWD	Gambian in Western Division – Mandinka	AFR	African

HG02667	GWD	Gambian in Western Division – Mandinka	AFR	African
HG02763	GWD	Gambian in Western Division – Mandinka	AFR	African
HG02772	GWD	Gambian in Western Division – Mandinka	AFR	African
HG02805	GWD	Gambian in Western Division – Mandinka	AFR	African
HG02837	GWD	Gambian in Western Division – Mandinka	AFR	African
HG02879	GWD	Gambian in Western Division – Mandinka	AFR	African
HG02891	GWD	Gambian in Western Division – Mandinka	AFR	African
HG03046	GWD	Gambian in Western Division – Mandinka	AFR	African
HG03539	GWD	Gambian in Western Division – Mandinka	AFR	African

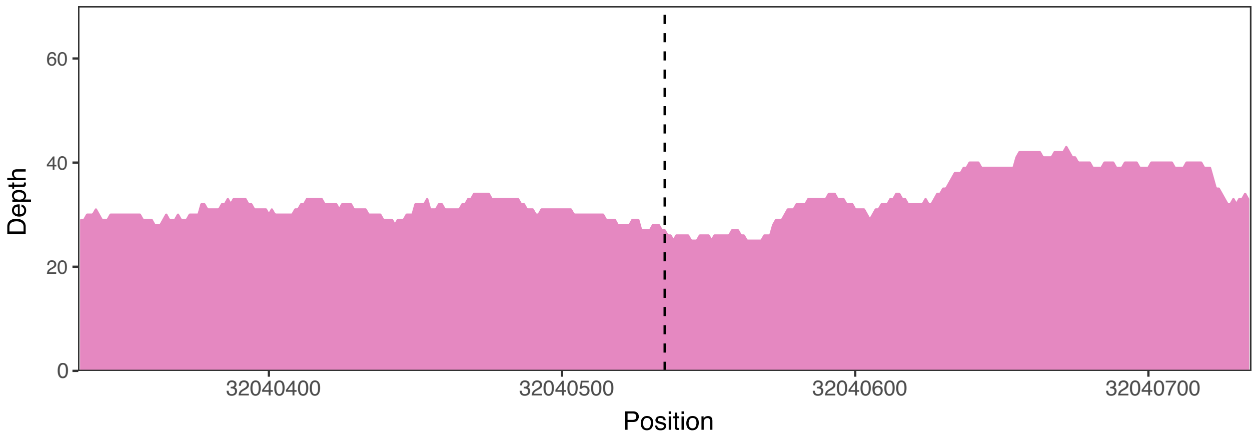

Supplementary Figure 1. Depth of mapping coverage of across *CYP21A2* when a homozygous pathogenic variant is inserted at position chr6:32040535 and 150bp reads are simulated with a 255bp inner distance.
