# Supplementary material for: Next-generation sequencing of newborn screening genes: the accuracy of short-read mapping
Source: NPJ Genom Med. 2020 Sep 4;5:36. doi: 10.1038/s41525-020-00142-z (PMC7474066; doi:10.1038/s41525-020-00142-z)
Supplement: Supplementary file 2 — Reporting Summary [file 41525_2020_142_MOESM2_ESM.pdf]

nature research

Corresponding author(s): Cassandra Trier

Last updated by author(s): 2020-18-7

Reporting Summary

Nature Research wishes to improve the reproducibility of the work that we publish. This form provides structure for consistency and transparency in reporting. For further information on Nature Research policies, see Authors & Referees and the Editorial Policy Checklist.

Statistics

For all statistical analyses, confirm that the following items are present in the figure legend, table legend, main text, or Methods section.

n/a	Confirmed	
☐	☒	The exact sample size (*n*) for each experimental group/condition, given as a discrete number and unit of measurement
☐	☒	A statement on whether measurements were taken from distinct samples or whether the same sample was measured repeatedly
☐	☒	The statistical test(s) used AND whether they are one- or two-sided<br>*Only common tests should be described solely by name; describe more complex techniques in the Methods section.*
☐	☒	A description of all covariates tested
☒	☐	A description of any assumptions or corrections, such as tests of normality and adjustment for multiple comparisons
☐	☒	A full description of the statistical parameters including central tendency (e.g. means) or other basic estimates (e.g. regression coefficient) AND variation (e.g. standard deviation) or associated estimates of uncertainty (e.g. confidence intervals)
☒	☐	For null hypothesis testing, the test statistic (e.g. *F*, *t*, *r*) with confidence intervals, effect sizes, degrees of freedom and *P* value noted<br>*Give P values as exact values whenever suitable.*
☒	☐	For Bayesian analysis, information on the choice of priors and Markov chain Monte Carlo settings
☒	☐	For hierarchical and complex designs, identification of the appropriate level for tests and full reporting of outcomes
☒	☐	Estimates of effect sizes (e.g. Cohen's *d*, Pearson's *r*), indicating how they were calculated

Our web collection on statistics for biologists contains articles on many of the points above.

Software and code

Policy information about availability of computer code

Data collection

A BED file of exonic positions for each gene in the panel was retrieved from GRCh38 on the Ensembl database Release 94 using biomaRt (v.3.8). FASTA sequences were retrieved for all exons in our gene panel using BEDTools (v.2.17.0) -getfasta function from GRCh38.p12 (RefSeq accession GCF_000001405.38). The 75 k-mer CGR Alignability track was downloaded from the UCSC depository (<http://rohsdb.cmb.usc.edu/>) in BigWig format and converted to a bed file with mappability values using UCSC's bigWigToWig tool followed by BEDOPS (v.2.4.35) wig2bed. Variant calls of 50 unrelated individuals from each of five super populations was downloaded from (<http://ftp.1000genomes.ebi.ac.uk/vol1/ftp/data_collections/1000_genomes_project/release/20181203_biallelic_SNV/>). A VCF file of human variants and disease associations was downloaded from ClinVar VCF (v.20181217).

Data analysis

A FASTA reference genome for each individual was created using bcftools (v.1.9) consensus with the VCF file for the individual and the GRCh38.p12 reference genome from the 1000 Genomes Project FTP site (ftp://ftp.1000genomes.ebi.ac.uk/vol1/ftp/technical/reference/GRCh38_reference_genome/). Illumina paired-end reads 70, 100, 150 and 250bp in length were simulated using DWGSIM (v.0.1.11) (https://github.com/nh13/DWGSIM) for each FASTA file of every individual.

The simulated reads were mapped to the GRCh38.p12 using BWA-MEM (v.0.7.17). Results from the read simulation were evaluated using the dwgsim_eval script provided by DWGSIM (https://github.com/nh13/DWGSIM). The depth of all simulated regions was calculated from the BWA output bam files using samtools depth (<http://www.htslib.org>).

Genotype likelihoods were calculated from each individuals' bam file using ANGSD (v.0.918). The covariance matrix of the genotype likelihoods was calculated using ngsTools' (v.3) ngsCovar tool. The PCA plot was created using the plotPCA.R script provided in ngsTools with the covariance file as input. Genetic differentiation between the simulated populations across NBS genes was calculated with ANGSD (v.0.918).

Consecutive low coverage bases were combined into a single larger region if they were within 50 bases of each other using BEDTools (v.2.17.0) merge. Regions were annotated using annotation information from the RefSeq GRC38.p12 annotation file (GCF_000001405.38_GRCh38.p12_genomic.fna.gz) using BEDOPS bedmap (2.4.35). The alternate regions that simulated reads mapped

to were extracted using the dwgsim_eval script. Each region was also annotated using BEDOPS bedmap (v.2.4.35) and was merged in the low coverage region bed file with the corresponding region it was simulated. The total number of reads for each low depth region, as well as the number of reads in each region that were not uniquely mapped were calculated using a custom script (available at https://github.com/cntrier/NBS_short-read_mapping_paper).

Sequence similarity was calculated by first running a MUSCLE pairwise alignment in Geneious (v. 2019.1.3) (https://www.geneious.com). The exported alignment file was plotted using AlignFigR (https://github.com/sjspielman/alignfigR) followed by custom alterations.

A VCF file was filtered for pathogenic variants within exonic regions of CYP21A2 using VCFtools (v.0.1.13). Using bcftools, each pathogenic variant VCF was applied to the reference FASTA to create a consensus FASTA with IUPAC coding. DWGSIM (v.0.1.11) (https://github.com/nh13/DWGSIM) was run separately for each homozygote and heterozygote FASTA sequence for every pathogenic variant with Illumina paired-end 150bp read lengths. Simulated reads were then mapped to the GRCh38.p12 human reference genome used previously with BWA MEM (v.0.7.17). Variant calling was performed on the processed reads with GATK (v.4.0) Haplotypecaller.

Custom code developed to run analyses in the study is available at https://github.com/cntrier/NBS_short-read_mapping_paper.

For manuscripts utilizing custom algorithms or software that are central to the research but not yet described in published literature, software must be made available to editors/reviewers. We strongly encourage code deposition in a community repository (e.g. GitHub). See the Nature Research guidelines for submitting code & software for further information.

Data

Policy information about availability of data

All manuscripts must include a data availability statement. This statement should provide the following information, where applicable:

- Accession codes, unique identifiers, or web links for publicly available datasets
- A list of figures that have associated raw data
- A description of any restrictions on data availability

The datasets generated during and/or analyzed during the current study are available at <https://github.com/cntrier/NBS_short-read_mapping_paper>. Generated mapped read files have been deposited in zenodo.org under the DOI 10.5281/zenodo.3950369. VCF files used for read simulation are publicly available from the 1000 Genome Project at <http://ftp.1000genomes.ebi.ac.uk/vol1/ftp/data_collections/1000_genomes_project/release/20181203_biallelic_SNV/>.

Field-specific reporting

Please select the one below that is the best fit for your research. If you are not sure, read the appropriate sections before making your selection.

☒ Life sciences ☐ Behavioural & social sciences ☐ Ecological, evolutionary & environmental sciences

For a reference copy of the document with all sections, see nature.com/documents/nr-reporting-summary-flat.pdf

Life sciences study design

All studies must disclose on these points even when the disclosure is negative.

Sample size	We simulated the genomes of 50 individuals with 10 individuals in each ethnic group to be able to compare between group variation. Our sample size allowed for analysis of data to answer our research questions while still being able to handle the data computationally.
Data exclusions	No data was excluded from analysis.
Replication	No experiments were performed.
Randomization	Grouping was based on ethnic population and read lengths, thus we did not need to perform any randomizations.
Blinding	As no observer scoring was performed in the study, we did not need any blinding.

Reporting for specific materials, systems and methods

We require information from authors about some types of materials, experimental systems and methods used in many studies. Here, indicate whether each material, system or method listed is relevant to your study. If you are not sure if a list item applies to your research, read the appropriate section before selecting a response.

Materials & experimental systems

n/a	Involved in the study
☒	☐ Antibodies
☒	☐ Eukaryotic cell lines
☒	☐ Palaeontology
☒	☐ Animals and other organisms
☒	☐ Human research participants
☒	☐ Clinical data

Methods

n/a	Involved in the study
☒	☐ ChIP-seq
☒	☐ Flow cytometry
☒	☐ MRI-based neuroimaging
